# Systematic annotation of orphan RNAs reveals blood-accessible molecular barcodes of cancer identity and cancer-emergent oncogenic drivers

**DOI:** 10.1016/j.xcrm.2025.102577

**Published:** 2026-01-23

**Authors:** Jeffrey Wang, Jung Min Suh, Brian J. Woo, Albertas Navickas, Kristle Garcia, Keyi Yin, Lisa Fish, Taylor Cavazos, Benjamin Hänisch, Daniel Markett, Gillian L. Hirst, Lamorna Brown-Swigart, Laura J. Esserman, Laura J. van ‘t Veer, Hani Goodarzi

**Affiliations:** 1 Department of Biochemistry & Biophysics, University of California, San Francisco, San Francisco, CA, USA; 2 Department of Urology, University of California, San Francisco, San Francisco, CA, USA; 3 Helen Diller Family Comprehensive Cancer Center, University of California, San Francisco, San Francisco, CA, USA; 4 Bakar Computational Health Sciences Institute, University of California, San Francisco, San Francisco, CA, USA; 5 Biological and Medical Informatics, University of California, San Francisco, San Francisco, CA 94158, USA; 6 Department of Surgery, University of California, San Francisco, San Francisco, CA 94143, USA; 7 Department of Laboratory Medicine, University of California, San Francisco, San Francisco, CA 94143, USA; 8 Arc Institute, Palo Alto, CA 94304, USA

**Keywords:** liquid biopsy, cancer biomarkers, RNA biology, oncogenic drivers, functional screen

## Abstract

From extrachromosomal DNA to neo-peptides, reprogramming of cancer genomes leads to the emergence of cancer state-specific molecules. Here, we systematically identify and characterize a large repertoire of orphan non-coding RNAs (oncRNAs), a class of cancer-emergent small RNAs, across 32 tumor types. We show that oncRNA binary presence-absence patterns represent a digital molecular barcode that captures cancer type and subtype identities. Importantly, this barcode is partially accessible from the cell-free space as cancer cells secrete a subset of oncRNAs. Leveraging large-scale *in vivo* genetic screens in xenografted mice, we functionally identify driver oncRNAs in multiple tumor types. In a retrospective study across 192 breast cancer patients, we show that oncRNAs are reliably detected in blood and that changes in cell-free oncRNA burden predict both short-term and long-term clinical outcomes. Together, we establish that oncRNAs have potential roles in tumor progression and clinical utility in liquid biopsies for tumor-naive minimum residual disease monitoring.

## Highlights

• Systematic annotation of orphan non-coding RNAs (oncRNAs) across 32 cancers
• OncRNAs patterns are molecular barcodes capable of distinguishing cancer types and subtypes
• Using large-scale genetic screens, we find some oncRNAs drive cancer progression
• Circulating oncRNAs are predictive of clinical outcomes in breast cancer patients

## Graphical abstract

**Figure:**
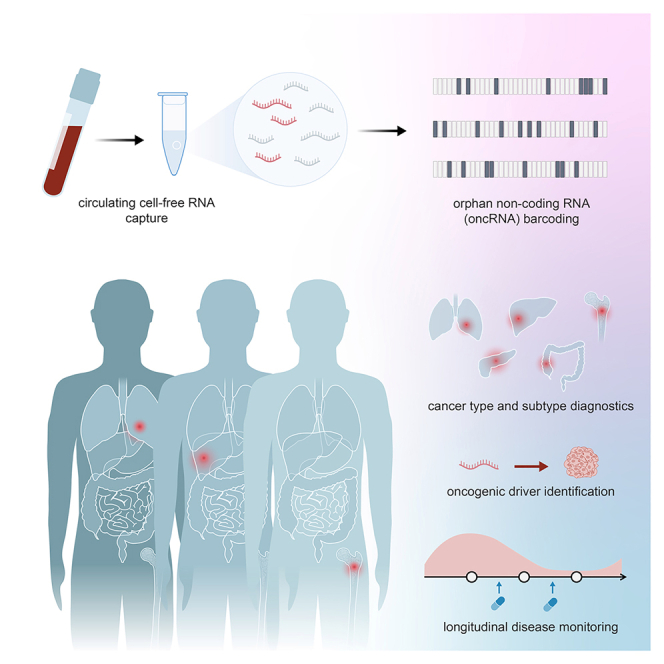

## Teaser

Wang et al. systematically annotate orphan non-coding RNAs (oncRNAs), cancer-emergent small RNAs, across 32 cancers. The study demonstrates that oncRNAs are informative of cancer types and have functional roles in cancer progression. Importantly, circulating oncRNAs are predictive of clinical outcomes in breast cancer patients, holding potential for liquid biopsy applications.

## Introduction

Cancer-emergent macromolecules, molecules that are uniquely present in cancer cells, have become the focus of many recent studies. Structural variations that lead to the expression of cancer-specific fusion proteins have long been known to play major roles in tumorigenesis.^1^^,^^2^^,^^3^ Tumors also generate cancer-specific neoantigens through the disruption of various cellular mechanisms.^4^^,^^5^ Extrachromosomal DNA is another class of cancer-emergent molecules that can drive oncogenesis.^6^^,^^7^ In breast cancer, we previously reported the discovery of orphan non-coding RNAs (oncRNAs), small non-coding RNAs that are expressed in cancer cells but absent in non-transformed tissue.^8^ We showed that one oncRNA derived from the TERC transcript plays a functional role in breast cancer metastasis by disrupting a miRNA-mRNA regulatory network controlling the expression of prometastatic genes.^8^ However, the extent to which oncRNAs may drive tumor progression across tumor types remains largely unexplored. In this study, we set out to systematically annotate oncRNAs across human cancers and discovered a large set of oncRNAs that are not only cancer-emergent but also cancer-specific. We then demonstrated the presence-absence patterns of oncRNAs in tumors represented digital molecular barcodes that can reliably discriminate between different cancer types and subtypes. Furthermore, we developed a large-scale *in vivo* genetic screening strategy to identify driver oncRNAs in multiple xenograft models of cancer. We discovered and subsequently validated several functional oncRNAs that impact tumor growth, highlighting their roles in disease progression.

We had previously shown that a fraction of oncRNAs is actively secreted by breast cancer cells and can potentially serve as a cancer-specific signal to distinguish serum samples from breast cancer and healthy patients. However, whether this signal was sufficiently strong to inform clinical practice in minimally invasive clinical applications was unknown. Here, we found that many annotated oncRNAs are also actively secreted across different cancers, implying that this oncRNA molecular barcode is partially blood-accessible and can provide an opportunity for a sensitive and versatile liquid biopsy strategy for multiple cancers. Minimal residual disease (MRD) monitoring in breast cancer via circulating tumor DNA (ctDNA) analysis is technically challenging, given the low ctDNA concentration during treatment, requiring tumor-informed assays to detect low tumoral variant frequencies.^9^ To assess the applicability of oncRNA-based liquid biopsies in MRD detection, we performed a large retrospective analysis of breast cancer patients in a neoadjuvant chemotherapy setting. We demonstrated that cell-free oncRNAs provide a tumor-naive strategy for MRD applications in breast cancer with minimal sample volume and limited sequencing depth. Altogether, our study encapsulates a comprehensive effort to annotate oncRNAs across human cancers and reveal their potential as digital biomarkers for cancer cell identity, functional macromolecules in cancer progression, and blood-accessible, prognostic biomarkers.

## Results

### Systematic annotation of orphan non-coding RNAs across human cancers

**Figure 1:**
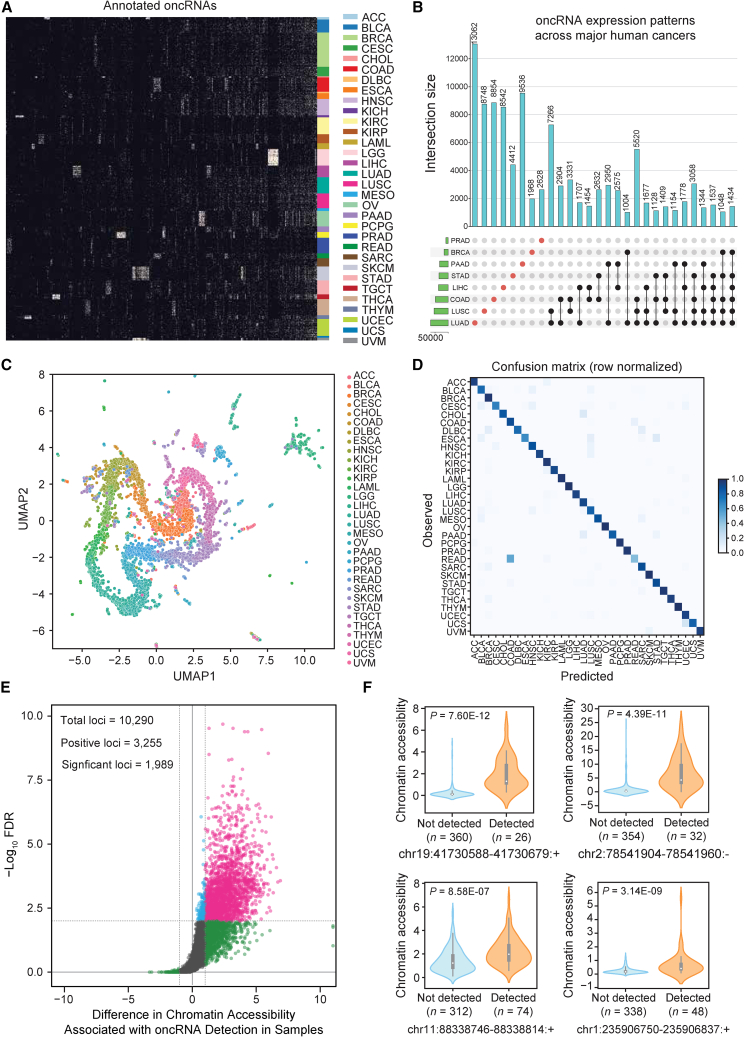
Systematic annotation of oncRNA loci across human cancers (A) A binary heatmap representing the presence (light beige) and absence (black) of oncRNA species across human cancers. Here, we show a subset of the top 2,808 significant oncRNAs. The subset was created by selecting 100 of the most significant oncRNAs for each cancer type as determined by the Fisher exact test and collapsing oncRNAs selected multiple times. Each column represents an oncRNA, and each row represents one TCGA sample. Rows were grouped based on tumor type, and columns were clustered based on their presence-absence patterns. (B) Number of oncRNAs associated with the major human cancers, namely lung, breast, and gastrointestinal cancers, depicted as an UpSet plot. Vertical blue bars represent the oncRNA counts across one or more cancers. (C) A 2D UMAP projection summarizing the oncRNA profiles across TCGA cancer samples. Samples are colored by tumor type. (D) Confusion matrix for tissue-of-origin classification by XGBoost classifier trained on binarized oncRNA expression data from TCGA training samples and evaluated on held-out TCGA cancer samples. The matrix was row-normalized. (E) A volcano plot representing the relationship between chromatin accessibility and oncRNA detection. The *x* axis represents, for each oncRNA, the log_2_ median difference in chromatin accessibility between samples in which the oncRNA was present versus absent. The *y* axis shows the significance of the observed differences based on FDR corrected *p* values calculated using a one-sided Mann-Whitney test. A total of 10,290 oncRNA loci were considered for this analysis based on the ATAC-seq coverage. Of these, 3,255 showed a positive association between oncRNA presence and increased chromatin accessibility; of these, 1,989 were also statistically significant at an FDR of 1%. (F) Chromatin accessibility signal of four exemplary oncRNA loci from (E), grouped by the detection of the cognate oncRNA in the small RNA dataset of each sample. Values are shown as violin plots and boxplots. The boxplots show the distribution quartiles, and the whiskers show the quartiles ± IQR (interquartile range). Also reported are the number of samples in which the oncRNAs were detected as well as their associated corrected *p* values from (E). See also Figure S1, Tables S1, S2, S3, S4, and S5.

To systematically discover and annotate oncRNAs, we analyzed small RNA (smRNA) sequencing data across 32 cancer types from The Cancer Genome Atlas (TCGA) dataset (see STAR Methods).^10^ Because TCGA lacks data from most blood cancers and non-cancerous biofluids, we also used smRNA sequencing data from non-cancerous samples in the Extracellular RNA Atlas (exRNA Atlas) to filter our annotations (Table S1).^11^ smRNA loci that were significantly present in cancer samples and absent in tumor-adjacent normal samples were annotated as oncRNAs. By applying this framework, we discovered 260,968 high-confidence oncRNAs that are specifically expressed in one or more cancers (Figures 1A and S1A). For example, we annotated 15,827 oncRNAs in breast cancer (TCGA-BRCA) and analyzed their presence and expression across both breast cancer and tumor-adjacent normal samples across all tissue types (Figures S1B and S1C). Overall, we annotated between 10^4^ and 10^5^ oncRNA species for each cancer type in TCGA (Figure S1D); some oncRNAs were unique to specific cancers while others were detected in more than one cancer (Figure 1B). Despite the low prevalence of any single oncRNA across all cancer samples (Figure S1E), we observed that the binary patterns of presence and absence of multiple oncRNAs, which we have named oncRNA barcodes, are readily distinguishable between cancer types. Comparing the median Jaccard similarity of oncRNA barcodes between samples from the same cancer tissue type versus all other cancer tissue types, we found significantly higher similarity among samples from the same tissue of origin (TOO) (Figure S1F). Therefore, each cancer type can be represented as a barcode based on the pattern of expressed oncRNAs (Figures 1A–1C and S1G).

To formalize this relationship, we took advantage of machine learning classifiers to assess the extent to which the oncRNA barcode from a given sample could be used to identify its TOO. For this task, we first split the samples from TCGA into train and test datasets (80:20 ratio). Within the training set, we used recursive feature elimination in a 5-fold cross validation setup to reduce the feature space (from 260,968 to 1,805 oncRNA features) and identify a robust set of oncRNAs to use as our molecular barcode for TOO prediction. We then trained an XGBoost classifier with 500 trees on this set of 1,805 oncRNAs on the whole training cohort. Applying the resulting model on the test data, we observed a strong performance with 90.9% accuracy. The performance metrics for each cancer are listed in Table S2, and the resulting confusion matrix reported in Figure 1D shows the fraction of correctly predicted samples by cancer type. Our model’s performance is comparable to gene-expression-based genetic algorithm/k-nearest neighbors and convolutional neural network classifiers, including the higher number of mistakes in distinguishing rectal adenocarcinomas (READ) from colon adenocarcinoma (COAD), which were also found in other studies to be biologically similar and often grouped together.^12^^,^^13^^,^^14^ Interestingly, we also found that our model’s errors were enriched with misclassifications between different squamous cancers (*p* = 1.24 × 10^−13^, Fisher exact test), including bladder urothelial carcinoma (BLCA), cervical squamous cell carcinoma (CESC), esophageal carcinoma (ESCA), head and neck squamous cell carcinoma (HNSC), and lung squamous cell carcinoma (LUSC), consistent with previously reported unsupervised clustering of different squamous tumors by various molecular platforms.^15^^,^^16^ This suggests that oncRNAs, like other previously reported molecular features including recurrent alterations in chromosomes, DNA methylation, and mRNA expression, are informative of the common underlying biology in squamous cell carcinomas. To emphasize the digital nature of oncRNA barcodes, we plotted the binary expression patterns of oncRNAs selected by the XGBoost classifier for TOO classification (Figure S1H). These results show that oncRNAs are cancer-informative and that our model can capture the heterogeneity of human cancers.

We also observed quantitative differences in the expression of oncRNAs beyond their binary presence-absence patterns, and thus asked whether including the relative oncRNA expression level could further improve our model’s TOO predictions (Figures S1I and S1J). To do this, we trained an XGBoost classifier on the counts-per-million (cpm) normalized oncRNA expression profiles, using an 80:20 ratio to split our samples into training and testing datasets. We found that the model trained on cpm data performed equally well and picked up important oncRNA features with similar patterns of expression as the binary model (Table S3; Figures S1K–S1N). The similarity in model performance of “digital” models trained on binarized oncRNA expression and “analog” models trained on normalized oncRNA expression data suggests that oncRNAs provide a digital barcode of cancer cell identity that is robust to the challenges in precise quantification of smRNAs.

Next, to confirm the reproducibility of our oncRNA annotations and digital barcodes in an independent cohort, we analyzed smRNA sequencing data from 938 tumors across six cancer types from the Clinical Proteomic Tumor Analysis Consortium (CPTAC).^17^ The cancer types analyzed include breast carcinoma (BRCA), colorectal cancer (CRC), lung adenocarcinoma (LUAD), lung squamous cell carcinoma (LUSC), pancreatic ductal adenocarcinoma (PAAD), and uterine corpus endometrial carcinoma (UCEC). Detailed cancer sample distributions are reported in Table S4. Remarkably, we found that 84.9% of our 260,968 annotated oncRNAs were detected in at least one cancer sample. We then trained an XGBoost classifier with 300 trees on the full set of binarized oncRNA expression data from 3,446 TCGA tumor samples of the corresponding six cancer types to predict TOO. The model was then evaluated on the 938 CPTAC tumor samples, achieving an overall accuracy of 82.1% (Table S5; Figure S1O). Our model’s robust performance in an independent test cohort further highlights the generalizability of an oncRNA digital barcode for encoding and accurately predicting cancer types.

Taken together, we have identified a large number of oncRNAs that are not only cancer-emergent but also reflective of cancer TOO. Two likely routes for these oncRNAs to emerge are (1) activation of cryptic promoters that lead to cancer-emergent transcriptional events and (2) aberrant nucleolytic digestion of longer RNAs. We previously described T3p, a breast-cancer-associated oncRNA derived from the *TERC* transcript, as an example of the latter pathway.^8^ Mapping our identified oncRNAs to their genomic locations suggests that 58.9% of oncRNAs may originate from existing longer RNAs. In contrast, the 41.1% of oncRNAs that map to intergenic regions are more likely produced by cancer-specific transcriptional activation (Figure S1P). To explore this hypothesis further, we used 386 assay for transposase-accessible chromatin using sequencing (ATAC-seq) samples from TCGA to compare chromatin accessibility between samples as a function of oncRNA expression across tumors.^18^ Approximately 10,000 intergenic oncRNA loci were captured at sufficient depth in the corresponding datasets. For a third of these loci, we observed a positive association between oncRNA expression in the small RNA data and chromatin accessibility in the ATAC-seq dataset, of which 1,989 oncRNA loci showed statistically significant associations at an FDR of 1% (Figure 1E). As expected, this association is entirely one-sided and we did not observe any oncRNAs in loci with closed chromatin. In Figures 1F and S1Q, we show the chromatin accessibility scores and relative expression of the top significant and expressed oncRNA loci as examples. To further validate our hypothesis of cancer-emergent transcriptional activity, we compared the chromatin accessibility of the significant oncRNA loci in previously published ATAC-seq data from the MDA-MB-231 breast cancer cell line.^19^ Notably, we found 1,204 (60.5%) oncRNA loci overlapped with ATAC-seq peaks. We also observed active transcription of 577 (29%) of the significant oncRNA loci in our previously published global run-on sequencing (GRO-seq) data from MDA-MB-231 cells.^20^ In Figure S1R, we show the GRO-seq coverage of three exemplary oncRNA loci. Together, these results strongly support our annotations and hypothesis that oncRNA biogenesis may arise from cancer-emergent transcription events in regions with increased chromatin accessibility.

### oncRNA expression patterns are associated with cancer subtypes

**Figure 2:**
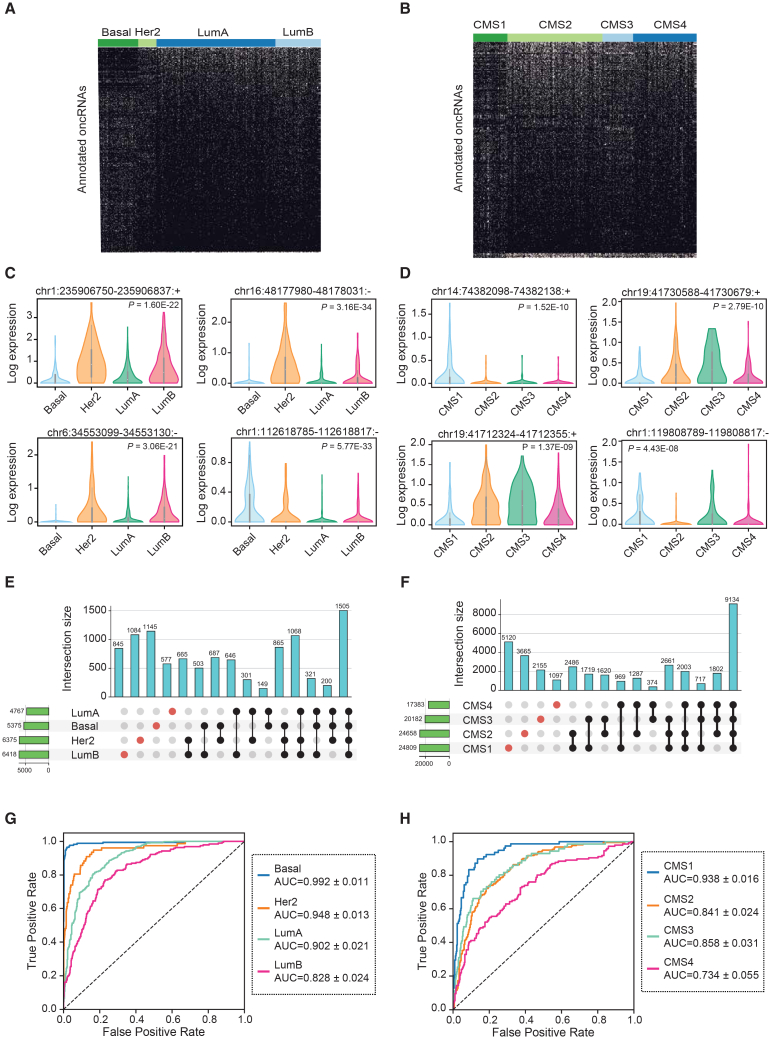
Annotation of subtype-associated oncRNAs across breast and colorectal cancer samples (A and B) Binary heatmaps of oncRNAs associated with breast cancer subtypes (A) and colorectal cancer CMS labels (B). One-way ANOVA tests followed by FDR correction were used to identify oncRNAs with significant associations. Each column represents a TCGA sample, and each row represents an oncRNA; oncRNA presence shown as light beige and absence as black. Columns were grouped by cancer subtype, and rows were clustered by their presence-absence patterns. (C and D) Exemplary subtype-associated oncRNA loci along with their expression patterns for breast cancer subtypes (C) or colon cancer CMS labels (D). The expression values are natural log transformed, and *p* values were calculated using a one-way ANOVA test. (E and F) The number of oncRNAs that were detected in one or more breast cancer subtypes (E) or colorectal cancer CMS labels (F) shown as UpSet plots. (G and H) ROC curves for XGBoost multiclass classifiers that predict the breast cancer subtype or colon cancer CMS label based on oncRNA presence/absence averaged across held-out validation sets in a 5-fold cross validation setup; 946 and 514 samples were tested in breast and colorectal cancer, respectively, and the resulting mean and standard deviation of AUCs were calculated for each subtype across the 5-folds. See also Figure S2.

In the previous section, we made two important observations: (1) oncRNAs show strong tissue-specific expression patterns, and (2) intergenic oncRNAs are associated with chromatin accessibility in cancer cells. Based on these findings, we hypothesized that oncRNA barcodes may reflect the cellular state of cancer cells. To assess this possibility, we sought to identify oncRNAs whose presence or absence were informative of cancer subtypes. For this purpose, we used the Prediction Analysis of Microarray 50 (PAM50) breast cancer subtype classification (i.e., basal, HER2+, and luminal A and B) as well as the consensus molecular subtype (CMS) framework in colon cancer.^14^^,^^21^ Following the CMS classification system methodology, we combined the TCGA-COAD and TCGA-READ cohorts into a single colorectal cancer (CRC) cohort for all subsequent analyses.^14^ Of the 15,827 breast-cancer-associated oncRNAs, 1,006 show significant subtype-specific patterns across the TCGA-BRCA cohort (Figure 2A). For the TCGA-CRC cohort, 1,198 of 57,632 CRC-associated oncRNAs demonstrate a significant association with CMS groups (Figure 2B). In Figures 2C and 2D, we also included the normalized expression of several oncRNAs significantly associated with tumor subtypes, highlighting the different quantitative patterns of expression across subtypes. Furthermore, we identified thousands of oncRNAs that were exclusively detected in samples of a given subtype for both breast and colorectal cancers, albeit insignificant when tested for subtype association across all samples (Figures 2E and 2F).

We then asked whether the cancer-associated oncRNAs could be leveraged to distinguish tumor subtypes using machine learning models. In a 5-fold cross-validation scheme, we used each training fold to train a multiclass XGBoost classifier. We then measured the performance of the model on the respective held-out fold. Breast cancer subtype classifiers achieved areas under the curve (AUCs) between 0.83 and 0.99; similarly, colon cancer CMS classifiers achieved AUCs ranging between 0.73 and 0.94 (Figures 2G and 2H). More detailed metrics of model performance for breast and colorectal cancers are reported in Figures S2A and S2B, respectively. Interestingly, we observed that the breast cancer models made a higher number of mistakes when distinguishing subgroups of luminal breast cancers, luminal A and B, which are known to be more closely related and harder to distinguish^22^ (Figure S2C). We did not observe any notable patterns of confusion for CMS classification (Figure S2D). We also show the binary patterns of all oncRNA features selected by the XGBoost classifier within each training fold across all samples and the relative expression of the oncRNAs with the top 10 average feature importance score (Figures S2E–S2H). Our results indicate that the XGBoost model is able to learn and leverage a subset of informative oncRNAs to accurately classify cancer subtypes for both breast and colorectal cancers. Together, these results further establish the utility of oncRNAs in not only distinguishing cancer TOO but also capturing their underlying cancer subtype identity.

### A systematic search for functional oncRNAs across multiple cancers

**Figure 3:**
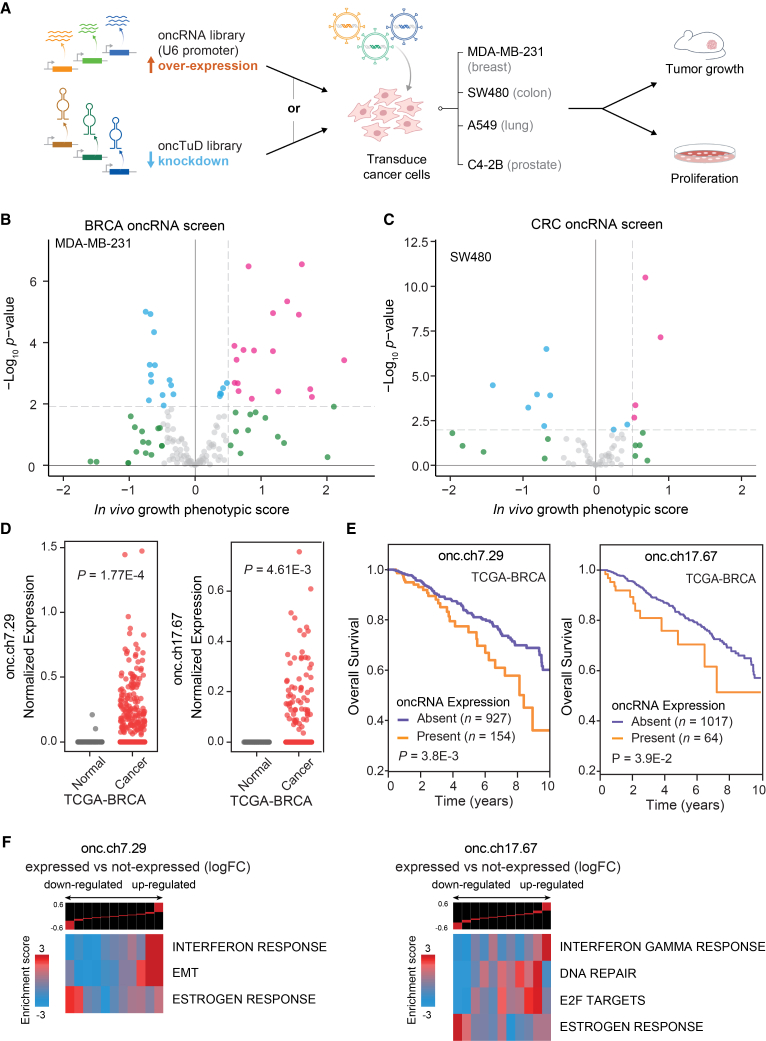
Systematic annotation of driver oncRNAs using a scalable *in vivo* genetic screening approach (A) Workflow schematic of oncRNA cancer and oncRNA TuD functional screens. (B and C) Volcano plots of oncRNA functional screen results for breast cancer (MDA-MB-231) and colorectal cancer (SW480), respectively. *In vivo* growth phenotypic score refers to the differential representation of cancer cells transduced with cognate oncRNA upon tumor growth in the xenograft model compared to the corresponding *in vivo* baseline. DESeq2 was used to compare *in vivo* and *in vitro* representation and calculate *p* values. Significance threshold was set at adjusted *p* value <0.05. (D) Expression levels of two example oncRNAs with significant tumor growth phenotype from the functional screen in TCGA-BRCA tumor and tumor-adjacent normal tissues. *p* values were calculated using a one-tailed Mann-Whitney test. (E) Survival of TCGA-BRCA patients stratified by presence or absence of the cognate driver oncRNA. *p* values were calculated using a log rank test. (F) Informative iPage pathways associated with TCGA-BRCA cancer samples expressing cognate oncRNAs compared to TCGA-BRCA cancer samples with no detectable respective oncRNAs. Top panel shows gene expression differences as log fold change (log FC) in discrete expression bins. Genes that are upregulated in oncRNA expressing cancer samples are in the right bins, whereas bins to the left contain genes with lower expression. The heatmap shows the corresponding pathway in relation to the expression bins. Red entries indicate enrichment of pathway genes in each expression bin, whereas blue entries indicate depletion. Enrichment and depletion are measured using log-transformed hypergeometric *p* values. See also Figure S3, Table S6.

Given the regulatory potential of oncRNAs through oncRNA-RNA or oncRNA-protein interactions, we had previously investigated the possibility that oncRNAs may be adopted by cancer cells to engineer cancer-specific regulatory pathways.^8^ Specifically, we uncovered one such oncRNA, T3p, and showed that it promotes breast cancer metastasis by dysregulating endogenous RISC complex activity. However, the extent to which other oncRNA species may play a functional role in cancer remains unexplored. The sheer number of oncRNA species emphasizes the need for systematic approaches to screen for functional representatives. To tackle this question, we developed a large-scale pooled *in vivo* screening framework to rapidly identify functional oncRNAs through gain- and loss-of-function studies as schematized in Figure 3A. For our gain-of-function study, we designed a library of lentiviral constructs encoding oncRNAs under the control of a U6 promoter to increase their expression. In parallel, we generated a lentiviral library of Tough Decoys (TuDs) to sequester oncRNAs and thereby achieve stable and sustained inhibitory effects for our loss-of-function study.^23^^,^^24^ We focused on four major cancers and selected a human cell line with an established xenograft model for each cancer: MDA-MB-231 (breast), SW480 (colon), A549 (lung), and C4-2B (prostate). We then used smRNA sequencing data from these cell lines to select expressed oncRNAs that were associated with each cell line’s respective tumor type in TCGA. For each cell line experiment, roughly 100 of the top expressed oncRNAs were selected for inclusion in the gain-of-function and loss-of-function (oncTuD) libraries. We also included non-targeting scramble sequences as endogenous controls. We transduced each of the four cell lines with their corresponding libraries and compared the representation of oncRNA/oncTuD species among cancer cell populations grown in mammary fat pads (MDA-MB-231) or subcutaneously (SW480, C4-2B, A549) *in vivo* (Figure S3A). For each oncRNA/oncTuD instance, we compared their normalized counts between *in vivo* grown tumors and *in vitro* controls to identify oncRNAs with changes in relative representation. We posited that changes in the baseline representation of cells harboring the cognate oncRNA/oncTuD lentiviral construct result from selection pressure during tumorigenesis, which we can use as a criterion to identify functional oncRNAs.

To identify oncogenic driver oncRNAs in our gain-of-function screens, we searched for those with increased expression in the tumors from xenografted mice. We discovered several candidate functional oncRNAs in the breast and colon cancer screens; however, the lung and prostate cancer screens did not nominate any significant oncRNAs (Figures 3B and 3C, S3B; Table S6). Similarly, for the oncTuD screens, we selected oncRNAs whose antisense TuDs showed a reduced representation in the tumors. As shown in Figures S3C and S3D and Table S6, several oncRNAs showed significant phenotypic effects within each cancer except for breast and lung cancer. Between the gain- and loss-of-function screens, roughly 5% of oncRNAs showed a significant tumor growth phenotype. This suggests that in addition to T3p, a subset of cancer-emergent oncRNAs likely play unexplored roles in disease progression across human cancers. Together, these findings establish a systematic means of nominating likely functional oncRNAs.

### Two breast-cancer-associated oncRNAs promote tumor growth and *in vivo* metastatic colonization

We next selected two exemplary breast cancer oncRNAs for deeper analysis of their function. In Figure 3D, we compared the normalized expression levels of these two oncRNAs between TCGA-BRCA cancer and tumor-adjacent normal tissue samples and demonstrated the highly cancer-specific expression pattern of these oncRNAs (referred to by their respective genomic coordinates oncRNA.ch7.29 and oncRNA.ch17.67). Both oncRNA.ch7.29 and oncRNA.ch17.67 map to the 3′ untranslated regions (UTRs) of cancer-associated genes, *SCRN1* and *PSMD12*, respectively.^25^^,^^26^ We also investigated the association of oncRNA expression with patient survival and found that these two oncRNAs were both significantly associated with poor clinical outcomes, further highlighting their potential clinical significance in breast cancer (Figure 3E). However, we did not find any significant associations when we stratified oncRNA expression by cancer stage or receptor subtype for either oncRNA (Figure S3E). To identify cellular processes that are associated with each oncRNA, we used the TCGA breast cancer dataset to compare the transcriptomic profiles between samples in which the oncRNA was detected versus absent. We performed differential gene expression analysis and found significant changes in the gene expression landscape of tumors expressing each oncRNA (Figure S3F). Subsequent pathway analysis using iPAGE revealed significant modulated pathways associated with each expressed oncRNA, raising the possibility that they are acting downstream of these functional oncRNAs to drive cancer progression (Figures 3F and S3G).^27^ Of note, we observed a significant association between oncRNA.ch7.29 expression and upregulation of genes in the epithelial-mesenchymal transition (EMT) pathway and significant associations between oncRNA.ch17.67 and upregulation of genes in the DNA repair and E2F pathways.

**Figure 4:**
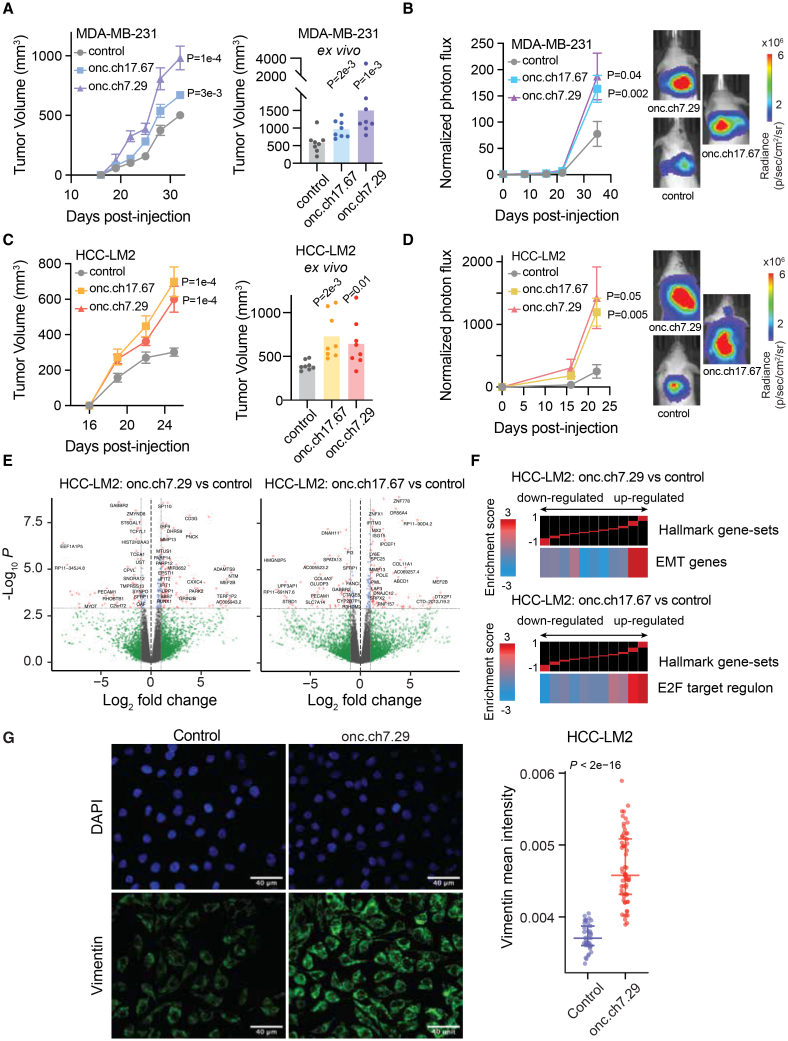
Two breast-cancer-associated oncRNAs promote tumor growth and *in vivo* metastatic colonization (A) Left: growth of MDA-MB-231 tumors overexpressing oncRNA.ch7.29 or oncRNA.ch17.67 relative to controls in the mammary fat-pad of NSG mice. Two tumors per mouse and *n* = 4 mice for each cohort. *p* values were calculated using two-way ANOVA. Right: *ex vivo* tumor measurements after tumor excision. *p* values were calculated using a one-tailed Mann-Whitney test. Data are represented as mean ± SEM. (B) Bioluminescence imaging plot of lung colonization by MDA-MB-231 cells overexpressing oncRNA.ch7.29 or oncRNA.ch17.67 compared to control. *n* = 5 per cohort. *p* values were calculated using two-way ANOVA. Data are represented as mean ± SEM. (C) Left: growth of HCC-LM2 cells overexpressing oncRNA.ch7.29 or oncRNA.ch17.67 and HCC-LM2 controls in the mammary fat-pad of NSG mice mammary fat-pad assays. *n* = 4 for each cohort. *p* values were calculated using two-way ANOVA. Right: *ex vivo* tumor measurements after tumor excision. *p* values were calculated using a one-tailed Mann-Whitney test. Tumors overexpressing oncRNA.ch7.29 were 1.6-fold larger than controls. Tumors overexpressing oncRNA.ch17.67 were 1.8-fold larger than controls. (D) Bioluminescence imaging plot of lung colonization by HCC-LM2 cells overexpressing oncRNA.ch7.29 or oncRNA.ch17.67 compared to control. *n* = 5 per cohort. *p* values were calculated using two-way ANOVA. (E) Volcano plots of differentially expressed genes in HCC-LM2 cells overexpressing oncRNA.ch7.29 or oncRNA.ch17.67 compared to HCC-LM2 controls. The *p* values were calculated using DESeq2 with cutoff corresponding to a 10% FDR. (F) Representative pathways associated with HCC-LM2 overexpressing oncRNA.ch7.29 or oncRNA.ch17.67 compared to controls generated using iPAGE. See Figure 3F legend for more details. (G) Immunofluorescence staining for vimentin (green) in control and onc.ch7.29-overexpressing breast cancer cell line, HCC-LM2. Top panels show DAPI (blue) signals. Vimentin intensity for control cells and onc.ch7.29-overexpressing cells were quantified using the raw images (*n* = 37 and 64, respectively). One-tailed Mann-Whitney *U* test was used to compare the measurements. Brightness of images were adjusted for visualization purposes only. Data are represented as mean ± SEM. Scale bar length corresponds to 40 μm. See also Figure S4.

We then performed *in vivo* tumor growth and metastasis assays to further validate the oncogenic role of these two oncRNAs. To test their effect, we first transduced MDA-MB-231 cells with oncRNA.ch7.29 or oncRNA.ch17.67 under the control of a U6 promoter for increased expression. Overexpression of oncRNA.ch7.29 and oncRNA.ch17.67 both significantly increased the primary tumor growth rates of cells implanted in the mammary fat-pad of NOD *scid* gamma (NSG) mice by 2.6- and 1.7-fold, respectively, relative to scrambled controls (Figure 4A). We then injected these transfected cells into the venous circulation of NSG mice and measured their lung metastatic colonization over time via bioluminescence imaging. Both oncRNA.ch7.29- and oncRNA.ch17.67-overexpressing cells had significantly increased capacity for lung colonization when compared to controls (Figures 4B and S4A). We repeated these experiments in an independent breast cancer cell line, metastatic derivative of HCC1806 genetic background (HCC-LM2^28^), to ensure that our observations were not cell line dependent. We found that HCC-LM2 cells overexpressing oncRNA.ch7.29 or oncRNA.ch17.67 also exhibited significantly higher primary tumor rates and metastatic capacity (Figures 4C, 4D, and S4A).

We next asked if the function of these oncRNAs was mediated through the same associated pathways we identified in TCGA-BRCA. To test this, we compared the transcriptomes of our cancer cells lines overexpressing oncRNA.ch7.29 or oncRNA.ch17.67 relative to controls in both genetic backgrounds (Figures 4E and S4B). Pathway analysis of differential expression patterns revealed modulations in key oncogenic pathways that were also observed in TCGA samples (Figures S4C and S4D), highlighting reproducible modulations of cellular pathways. Specifically, overexpressing oncRNA.ch7.29 resulted in increased expression of EMT-related genes, consistent with our observations in TCGA-BRCA tumors expressing oncRNA.ch7.29 (Figures 4F and 3F). We then performed immunofluorescence staining for vimentin, a canonical EMT marker, in our oncRNA.ch7.29-overexpressing and control breast cancer cell lines. Remarkably, we observed a significant upregulation of vimentin in both HCC-LM2 and MDA-MB-231 cell lines overexpressing oncRNA.ch7.29, confirming our pathway analysis findings (Figures 4G and S4E). These results strengthen oncRNA.ch7.29’s role in promoting cancer progression by mediating epithelial-mesenchymal transition in cancer cells.

Likewise, oncRNA.ch17.67-overexpressing cells demonstrated perturbation of the E2F pathway in a similar pattern as TCGA-BRCA tumors expressing oncRNA.ch17.67 (Figures 4F and 3F). While many significant oncRNA-associated pathways were shared among the HCC-LM2 and MDA-231 genetic backgrounds, we note that the E2F target regulon was not shown to be significantly associated with oncRNA.ch17.67 in MDA-231 cells (Figure S4D). Together, our findings strongly support that a subset of oncRNAs drive oncogenesis, likely by perturbing specific gene pathways.

### Annotation of cell-free orphan non-coding RNAs across models of cancer

**Figure 5:**
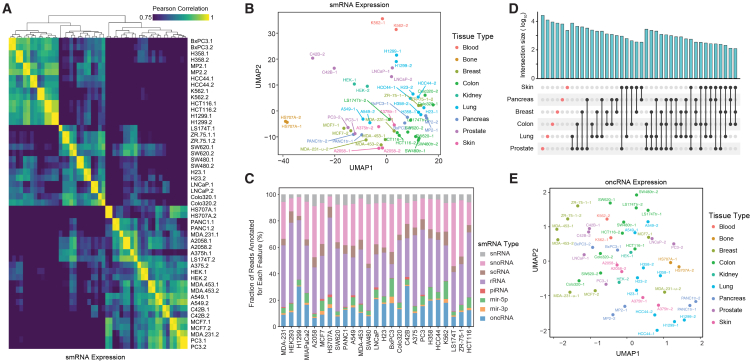
Analysis of cell-free RNA content across a large panel of cancer cell lines (A) Pairwise correlation heatmap for smRNA abundance in the cfRNA extracted from conditioned media. The counts for annotated smRNAs, such as miRNAs, tRNA fragments, snoRNAs, were used to generate this heatmap. (B) A 2D UMAP plot summarizing the abundance of smRNAs in the cell-free space across the cell line models we have profiled (in biological replicates). The points are colored based on the tissue of origin. (C) Contribution of each annotated family of smRNA species to their cfRNA content relative to annotated RNAs, omitting cfRNA with no known annotations. The values are normalized across cell lines, and oncRNAs are shown in blue. (D) An UpSet plot of oncRNA counts detected in the cfRNA fraction of cell lines from each tissue of origin. Cell-free oncRNAs show tumor-specific patterns of expression. (E) 2D UMAP summary of cell-free oncRNA profiles across tumor cell lines. See also Figure S5.

We had previously observed that a subset of breast cancer oncRNAs are secreted from breast cancer cells at detectable levels in the extracellular space.^8^ To investigate whether secreted oncRNAs are generalizable to other cancer types, we selected 25 established human cancer cell lines representing nine TOOs—blood, bone, breast, colon, kidney, lung, pancreas, prostate, and skin. After growing the cell lines *in vitro*, we collected conditioned media with exosome-depleted fetal bovine serum (FBS) in biological replicates, extracted RNA from the cell-free conditioned media, and performed smRNA sequencing. Many smRNAs, such as microRNAs, YRNAs, and tRNA fragments are known to be secreted into the extracellular space.^29^^,^^30^^,^^31^^,^^32^ As shown in Figures 5A, 5B, and S5A, annotated smRNA profiles from biological replicates cluster together and, overall, cell lines from the same TOO show similar patterns. We used this dataset of cell-free RNA (cfRNA) content to identify oncRNAs that are expressed and secreted from each cell line. Overall, we observed cell-free smRNA reads mapping to thousands of oncRNA loci, making this biotype a significant contributor to the extracellular smRNA space relative to other biotypes (Figure 5C). Roughly 0.5% of cfRNA reads were annotated as oncRNAs in our pipeline, with about 30% of our pancancer list of oncRNAs detected in at least two cell lines (Figure S5B). Similar to our observation in tumor biopsies, we observed tumor-type-specific oncRNAs among the cell-free oncRNAs (Figure 5D). Furthermore, 2D UMAP and PCA visualizations of cell-free oncRNA profiles show clustering of cell lines by TOO (Figures 5E and S5C). To quantify this similarity, we compared the median correlation between each cell line’s oncRNA profile with those from cell lines of the same tissue versus all other cell lines. Consistently, we observed a higher correlation between cell lines from the same TOO than those from different TOOs (Figure S5D). Taken together, our analysis demonstrates that oncRNAs contribute to the cfRNA content of cancer cells and that cell-free oncRNA expression profiles reflect tumor-type-specific patterns.

### Circulating oncRNAs capture short-term and long-term clinical outcomes in breast cancer

**Figure 6:**
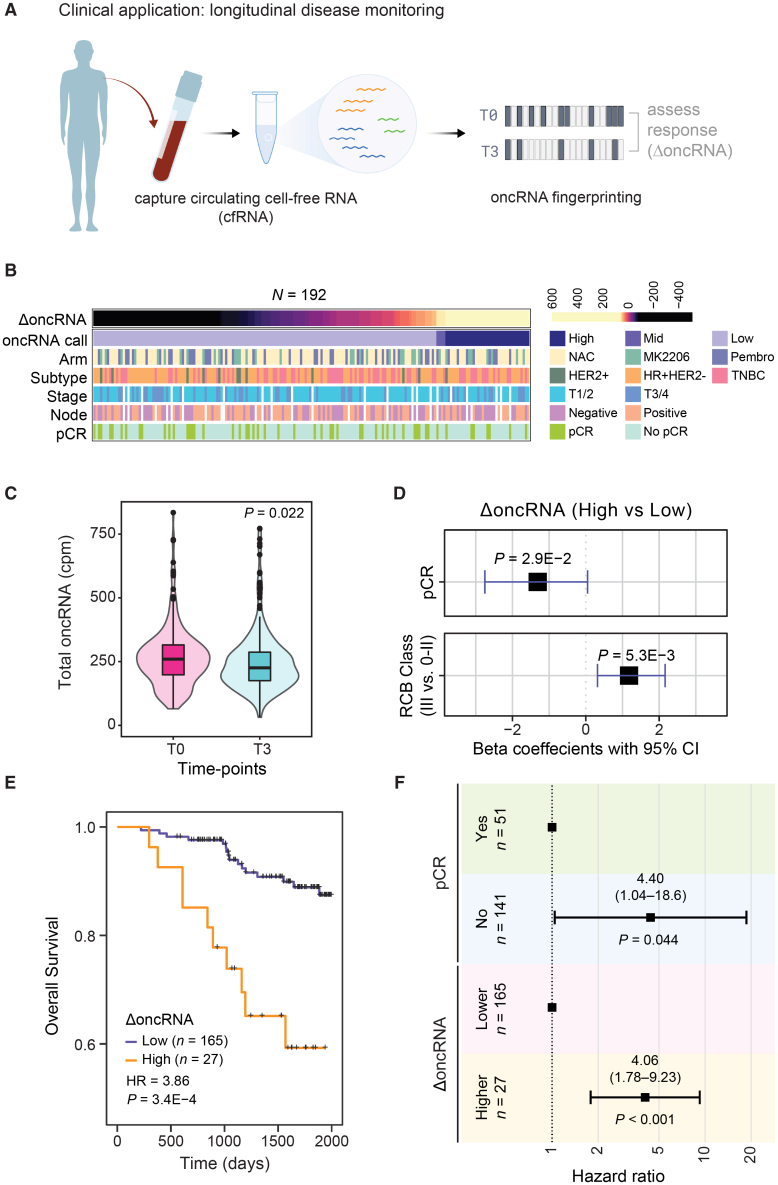
Changes in circulating oncRNA content over the course of neoadjuvant chemotherapy are informative of short-term and long-term clinical outcomes (A) Workflow schematic of oncRNA-based liquid biopsy for minimum residual disease detection. (B) Overview of patient and tumor characteristics tabulated based on changes in oncRNA burden (ΔoncRNA). (C) Normalized oncRNA burden (cpm) before (T0) and after (T3) NAC. *p* value was calculated using a one-tailed Wilcoxon test. Values are shown as violin plots and boxplots. The boxplots show the distribution quartiles, and the whiskers show the quartiles ± IQR (interquartile range). (D) Forest plots for logistic regression models predicting pathologic complete response (pCR) or high residual cancer burden (RCB III) as a function of ΔoncRNA after NAC. One-tailed *p* values are also included. (E) Survival in patients grouped based on their ΔoncRNA. Reported are the hazard ratio and *p* value based on a log rank test. (F) A forest plot for a multivariate Cox proportional hazard model including both ΔoncRNA and pCR as covariates. *p* values from the multivariate Cox analysis for each covariate are also included. See also Figure S6.

Thus far, we have established that cell-free oncRNAs faithfully reflect cancer type identity. Since oncRNAs are cancer-emergent, their presence in circulating blood points to the presence of an underlying tumor that is actively releasing them. This notion is supported by our previous work showing that circulating oncRNA can be used to detect breast cancer in patients.^8^ To assess the clinical utility of circulating oncRNAs as a cancer-specific biomarker, we performed a retrospective study on longitudinally collected samples from high-risk early breast cancer patients enrolled in the multicenter I-SPY 2 TRIAL (NCT01042379).^33^ We extracted cfRNA from 1 mL serum samples from 267 breast cancer patients treated with standard neoadjuvant chemotherapy (NAC) alone or combined with MK-2206 (AKT inhibitor) or Pembrolizumab (PD-1 inhibitor) treatment. For each patient, we processed longitudinal serum samples collected at pretreatment (T0) and post-NAC and prior to surgery (T3) for smRNA sequencing. For 192 patients with T0 and T3 samples that passed our quality control filters, we measured total oncRNA burden, defined as the sum of all oncRNA species across all loci normalized by library size, for each time point. We then used the change in oncRNA burden before and after treatment (ΔoncRNA) as a measure of residual oncRNA burden, schematized in Figure 6A. Detailed descriptions of our final patient cohort in our analysis are summarized in Figures 6B and S6A. In Figure S6B, we report the distribution of the resulting residual oncRNA burden classes across cancer subtypes, stages, and node status. Consistent with the response to treatment in most patients, we observed a significant overall reduction in oncRNA burden after NAC (Figures 6C and S6C).

Short-term clinical responses to NAC, i.e., pathologic complete response (pCR) and residual cancer burden (RCB) class, are strongly associated with favorable outcomes in the I-SPY 2 trial. Thus, we first examined whether our ΔoncRNA calls were associated with these early clinical readouts. We used logistic regression to assess the association between residual oncRNA burden after NAC with pCR and RCB classification, respectively. As shown in Figure 6D, in both cases, we observed a significant association between ΔoncRNA and short-term clinical responses.

With a median follow-up of 4.72 years in our study, we next sought to measure the extent to which residual oncRNA burden captures long-term clinical outcomes. For both overall survival and disease-free survival, we observed that high ΔoncRNA is significantly associated with poor survival outcomes (Figures 6E and S6E). These associations were not highly sensitive to the choice of threshold for the high ΔoncRNA call in patients (Figure S6F). Finally, we asked whether residual oncRNA burden provided additional information over pCR and RCB class regarding long-term survival. For this, we performed multivariable Cox regression analyses, and we observed that ΔoncRNA remains significantly informative of survival even when controlling for pCR or RCB (Figures 6F and S6G). Residual oncRNA burden also provided additional information when we controlled for tumor subtype and patient age (Figure S6H), highlighting the added benefit of disease monitoring via oncRNA burden dynamics. Together, these findings establish circulating oncRNAs as clinically relevant liquid biopsy biomarkers that can be accessed from low volumes of blood.

## Discussion

In this study, we discovered and systematically annotated a class of cancer-specific RNA species, oncRNAs, which have largely remained unexplored in the context of cancer biology. Our analysis not only reveals that these oncRNAs exhibit remarkable cancer type and subtype specificity but also highlights the possible functional roles of oncRNAs for cancer progression. Leveraging our *in vivo* screening platform, we revealed that a small subset of oncRNAs significantly impacts tumor growth phenotypes. Importantly, we view these numbers of significant hits to be a conservative estimate due to several factors. First, the lentiviral constructs described here are not guaranteed to up- or downregulate their cognate oncRNAs: RNA-polymerase-III-driven exogenous oncRNAs may be more unstable than endogenously expressed and processed oncRNAs, and TuDs may insufficiently inhibit their target oncRNAs, requiring fine-tuning of TuD design (i.e., optimizing thermodynamic properties) for adequate potency.^34^ Second, functions of oncRNAs are likely context-dependent, and the inclusion of other xenograft models will likely yield additional functional species. Finally, xenograft models only capture some aspects of tumor growth, lacking key characteristics such as adaptive immunity and native tumor microenvironment. Our findings establish a systematic approach of combining *in vivo* screens and computational analysis to nominate oncRNA drivers of oncogenesis. Namely, we consider oncRNAs that (1) display cancer-specific expression in both TCGA tumors and cancer cell line models, (2) present phenotypic effects in our functional screens, (3) demonstrate significant associations with poor clinical outcomes, and (4) are associated with cancer-relevant gene pathways as prime candidates for further functional investigations.

Although the molecular mechanism of action and biogenesis of oncRNA.ch7.29 and oncRNA.ch17.67 remain unknown, this study substantially expands our catalog of cancer-engineered oncogenic pathways and opens avenues for exploring oncRNAs as therapeutic targets in cancer. Specifically, we found oncRNA.ch7.29 and oncRNA.ch17.67 to be significantly associated with modulations in EMT and E2F pathways, respectively. EMT is a crucial hallmark for cancer progression, particularly through loss of cell adhesion, resistance to apoptosis, and acquired invasiveness.^35^ While non-coding RNAs like miRNAs have been shown to regulate cancer cell invasion and metastasis by targeting the mRNA of EMT-inducing transcription factors, our results suggest that cancer cells can also co-opt the complex EMT process via cancer-emergent RNA species.^36^^,^^37^^,^^38^ The E2F target regulon collectively controls cell-cycle progression and is commonly activated in cancer cells to drive tumor proliferation.^39^ Consequently, there has been much attention for therapeutic interventions that affect E2F activity via targeting the CDK-RB-E2F axis through CDK4/6 inhibitors for breast cancer.^40^ oncRNA.ch17.67’s upregulation of E2F genes may partially explain the increased tumor proliferation rate observed in our xenograft models and present as another potential therapeutic target to control E2F’s activity. Given E2F’s non-canonical role in apoptosis, metabolism, and angiogenesis, oncRNA.ch17.67 may also promote metastasis in a cell-proliferation-independent manner.^39^^,^^41^ Because oncRNAs are largely absent in normal cells, targeting these cancer-associated pathways via oncRNAs may offer a specific therapeutic advantage by minimizing off-target toxicity and therefore reducing patient side effects.

Most importantly, our study shows that oncRNAs can be reliably detected in the circulating blood of cancer patients, making them valuable biomarkers for clinical applications. The current state-of-the-art liquid biopsy strategies for minimal residual disease detection in breast cancer rely on development of tumor-informed bespoke assays for detection of high variant allele frequency (VAF) mutations in the blood.^42^^,^^43^ Due to low DNA shedding from breast tumors, however, even with these bespoke assays, DNA-based modalities are often not sensitive enough to reliably detect residual disease after clinical intervention.^42^ Circulating oncRNAs allow us to overcome these limitations for liquid biopsy markers. The much larger feature space of oncRNAs confers higher robustness against the zero-inflated nature of circulating biomarkers. Additionally, cancer cells actively secrete RNA, whereas DNA is passively shed because of cell death.^44^ Thus, cell-free RNA biomarkers are often more abundant than their DNA counterparts, making oncRNAs highly sensitive biomarkers that can be detected even in low volumes of blood after treatment. Furthermore, detecting circulating oncRNAs preclude the need to profile patients’ primary tumors, providing a tumor-naive approach to monitoring cancer. Other cell-free RNAs, including microRNAs, repeat element-derived RNAs, and tRNA fragments, have also been of recent research interest for their potential as circulating biomarkers of cancer.^45^^,^^46^^,^^47^^,^^48^ While prior studies have shown cfRNA profiles to be promising for applications in cancer detection, cfRNA signatures have primarily been discovered directly from human plasma samples and are unlikely to be directly representative of the underlying tumor biology. These signatures also predominantly rely on RNAs of known annotations that can originate from any cell and may not be directly secreted by cancer cells. Furthermore, investigations of cfRNAs as clinical biomarkers have largely been restricted to applications in cancer detection with limited success.

In our retrospective study, we investigated the utility of circulating oncRNAs for MRD detection and predicting clinical outcome in a neoadjuvant chemotherapy setting. We combined all oncRNA species to define an oncRNA burden score and found the dynamic changes in the oncRNA burden score in response to NAC to be strongly associated with both short-term and long-term clinical outcomes. These results establish oncRNAs as biomarkers for minimally invasive and real-time monitoring of underlying cancers, which can significantly help guide cancer management. We anticipate that future liquid biopsy studies with substantially larger cohort sizes as well as larger collected blood volumes and deeper sequencing of the cfRNA content will enable us to delve deeper into the wealth of information offered by oncRNAs and potentially reveal cancer-subtype signatures, cancer subtype switching occurrences, or relationships to treatment response. As we continue to investigate the various roles and information carried by individual oncRNAs, we anticipate that these RNA species will prove to be invaluable tools in the ongoing battle against cancer.

### Limitations of the study

One of the primary challenges of identifying cancer-emergent and cancer-specific biomolecules stem from dataset-dependent noise that can be mistaken as true biological signal. To address these limitations and potential for dataset-dependent results, we (1) utilized an independent compendium of data from the exRNA Atlas to filter false-positive oncRNA calls; (2) validated our cancer TOO predictive model in CPTAC, another independent cohort of cancer samples; (3) investigated oncRNAs with orthogonal modality of sequencing (ATAC-seq); and confirmed the presence of oncRNAs in the (4) cell-free space of cancer cell lines and (5) circulating serum of breast cancer patients. Furthermore, we have also shown that two of our oncRNAs demonstrated phenotypic effects in our models of breast cancer progression. Together, these results strongly support our oncRNA annotations as generalizable and biologically and clinically relevant.

## Resource availability

### Lead contact

Requests for further information, resources, and reagents should be directed to and will be fulfilled by the lead contact, Hani Goodarzi (hani.goodarzi@ucsf.edu).

### Materials availability

All unique/stable reagents generated in this study are available from the lead contact with a completed materials transfer agreement.

### Data and code availability

• Sequencing data generated for this study were deposited to ArrayExpress under the accession number: E-MTAB-16091.
• Custom code used in this study can be found here: Github: https://github.com/goodarzilab/Pan-oncRNA. The corresponding DOI is Zenodo: https://doi.org/10.5281/zenodo.17519718.
• Any additional information required to reanalyze the data reported in this work paper is available from the lead contact upon request.

## Acknowledgments

We thank April Pawluk, Brian Plosky, and Chiara Ricci-Tam for their valuable feedback on earlier versions of this manuscript and assistance with graphics design. We thank Shaorong Yu for her assistance with RNA extraction. We thank Amy L. Delson and Carol Simmons, our patient advocates, for their valuable feedback throughout this study. We thank Christopher Carpenter for his assistance with processing sequencing data. The I-SPY2 trial is supported by the study sponsors, Quantum Leap Healthcare Collaborative (2013 to present) and a grant from the 10.13039/100000054National Cancer Institute (P01CA210961). Sequencing was performed at the UCSF CAT, supported by UCSF PBBR, RRP IMIA, and 10.13039/100000002NIH 1S10OD028511-01 grants. Additional support was provided by Friends for Breast Cancer, AACR, Emerson Collective, and Mark Foundation. H.G. is an Arc Core Investigator, and research in this paper was partly funded by the 10.13039/100032560Arc Institute. H.G. is an Era of Hope Scholar (W81XWH-2210121) and supported by grants from 10.13039/100000054NCI (R01CA240984 and R01CA244634). This work was also supported by Earlier, the 10.13039/100019329Emerson Collective, and the 10.13039/100014599Mark Foundation.

## Author contributions

H.G. conceived the study. J.W., J.M.S., B.J.W., A.N., L.F., and H.G. designed and performed experiments. J.W., T.C., and H.G. performed computational analyses. K.G., K.Y., B.H., and D.M. performed RNA isolations and prepared smRNA-seq libraries. G.L.H., L.B.-S., L.J.E., and L.J.v.V. contributed data from the I-SPY2 trial. J.W., J.M.S., B.J.W., and H.G. wrote and edited the manuscript. H.G. supervised the project.

## Declaration of interests

H.G. is a co-founder and shareholder of Exai Bio. J.W., L.F., and T.C. are employees and shareholders of Exai Bio. L.J.E. reports funding from Merck & Co.; participation on an advisory board for Blue Cross Blue Shield; and personal fees from UpToDate. L.J.v.V. is a founding advisor and shareholder of Exai Bio, part-time employee, and owns stock in Agendia. H.G. is an inventor on a patent related to this work.

## STAR★Methods

### Key resources table

**Table:** 

REAGENT or RESOURCE	SOURCE	IDENTIFIER
**Antibodies**		

Rabbit Anti-Vimentin antibody [EPR3776] - Cytoskeleton Marker	Abcam	Cat#ab92547; RRID: AB_10562134
Rabbit Anti-E Cadherin antibody [EP700Y] - Intercellular Junction Marker	Abcam	Cat#ab40772; RRID: AB_731493
Goat anti-Rabbit IgG (H + L) Highly Cross-Adsorbed Secondary Antibody, Alexa Fluor™ 488	ThermoFisher Scientific	Cat#A-11034; RRID: AB_2576217
Human IgG (Fc) Polyclonal - Isotype Control	Abcam	Cat#ab206214

**Bacterial and virus strains**		

MegaX DH10B T1R electrocompetent cells	Invitrogen	Cat. #C640003

**Biological samples**		

Serum samples from human patients	ISPY-2 Clinical Trial	N/A

**Chemicals, peptides, and recombinant proteins**		

FD Esp3I	Thermo Fisher	Cat. #FD0454
AgeI-HF	NEB	Cat. #R3552S
EcoRI-HF	NEB	Cat. #R3101S
Penicillin-Streptomycin-Glutamine (100×)	Thermo Fisher	Cat#10378016
Amphotericin B	Thermo Fisher	Cat#30-003-CF
Corning Matrigel Basement Membrane Matrix, LDEV-free	Corning	Cat#354234
D-luciferin	Revvity	Cat#122799

**Critical commercial assays**		

DNA Clean and Concentrator kit-5	Zymo Research	Cat. #D4003
MinElute PCR purification kit	Qiagen	Cat. #28004
Select-a-Size DNA Clean & Concentrator MagBead Kit	Zymo	Cat. #D4084
MiSeq v2	Illumina	Cat. #MS-102-2002
Quick-DNA midiprep plus kit	Zymo Research	Cat. #D4075
QuantSeq 3′ mRNA-Seq Library Prep Kit FWD	Lexogen	Cat. #015
SMARTer® smRNA-Seq Kit for Illumina	Takara Bio	Cat#635031
*Quick*-cfRNA Serum & Plasma Kit	Zymo Research	Cat#R1059

**Deposited data**		

mRNA-seq	In this paper	Accession: E-MTAB-16091

**Experimental models: Cell lines**		

MDA-MB-231	ATCC	Cat#HTB-26
SW480	ATCC	Cat#CCL-228
A549	ATCC	Cat#CCL-185
C4-2B	ATCC	Cat#CRL-3315
MDA-MB-231 shctrl	This paper	N/A
HCC1806 shctrl	This paper	N/A
MDA-MB-231 oncRNA.ch7.29	This paper	N/A
HC1806 oncRNA.ch7.29	This paper	N/A
MDA-MB-231 oncRNA.ch17.67	This paper	N/A
HCC1806 oncRNA.ch17.67	This paper	N/A
MCF7	ATCC	Cat#HTB-22
A375	ATCC	Cat#CRL-1619
LS174T	ATCC	Cat#CL-188
PANC-1	ATCC	Cat#CRL-1469
MDA-MB-453	ATCC	Cat#HTB-131
Hs 707(A).T	ATCC	Cat#CRL-7448
HEK293	ATCC	Cat#CRL-1573
A2058	ATCC	Cat#CRL-3601
HCC-44	DSMZ	Cat#ACC534
NCI-H23	ATCC	Cat#CRL-5800
NCI-H1299	ATCC	Cat#CRL-5803
NCI-H358	ATCC	Cat#CRL-5807
BxPC-3	ATCC	Cat#CRL-1687
LNCaP	ATCC	Cat#CRL-1740
ZR-75-1	ATCC	Cat#CRL-1500
K-562	ATCC	Cat#CCL-243
MIA PaCa-2	ATCC	Cat#CRL-1420
COLO320DM	ATCC	Cat#CCL-220
HCT116	ATCC	Cat#CCL-247
SW620	ATCC	Cat#CCL-227
PC-3	ATCC	Cat#CRL-1435

**Experimental models: Organisms/strains**		

NOD.Cg-*Prkdc*^*scid*^*Il2rg*^*tm1Wjl*^/SzJ	Jackson Laboratory	Strain#005557

**Oligonucleotides**		

oncTuD library amplification F:ATTTTGCCCCTGGTTCTT	IDT	N/A
oncTuD library amplification R:CCCTAAGAAATGAACTGG	IDT	N/A
oncTuD amplicon-F: GGAAAGGACGAAACACCGGT	IDT	N/A
oncTuD amplicon-R: ATACTGCCATTTGTCTCGAGGTC	IDT	N/A
oncTuD amplicon-F-2: ACACTCTTTCCCTACACGACGCTCTTCCG ATCTGGAAAGGACGAAACACCGGT	IDT	N/A
oncTuD amplicon-R-2: GTGACTGGAGTTCAGACGTGTGCTCTTCCGA TCTATACTGCCATTTGTCTCGAGGTC	IDT	N/A
Illumina TruSeq UDI index set	Illumina	N/A
oAN491 (custom TSO oligo with UMI used with Takara smRNA kit)	IDT	N/A
oAN426 (custom cDNA amplification primer R, used with Takara smRNA kit)	IDT	N/A
oAN425 (example of custom cDNA amplification primer F, used with Takara smRNA kit	IDT	N/A

**Recombinant DNA**		

pUC6	Addgene	Cat #49793
pLKO.1	Addgene	Cat #10878
pLKO.1 shctrl	This paper	N/A
pLKO.1 oncRNA.ch7.29	This paper	N/A
pLKO.1 oncRNA.ch17.67	This paper	N/A

**Software and algorithms**		

Pan-oncRNA	Github: https://github.com/goodarzilab/Pan-oncRNA	Zenodo: https://doi.org/10.5281/zenodo.17519718

### Experimental model and study participant details

#### Cell lines and cell culture

All cell lines were maintained at 37°C in a humidified incubator with 5% CO2. All culture media (Gibco) were supplemented with 10% FBS (Corning), 100 units/mL penicillin, 100 μg/mL streptomycin (Gibco), and 1 μg/mL amphotericin B (Gibco). Cell lines were cultured in the following media: DMEM - MCF7, A375, LS174T, PANC-1, MDA-MB-231, MDA-MB-453, C42B, HS707A, HEK293, and A2058; RPMI 1640 - HCC44, NCI-H23, NCI-H1299, NCI-H358, BxPC-3, LNCaP, ZR-75-1, K562, MIA PaCa-2, Colo320, and HCC1806-LM2; McCoy’s 5A - HCT116, SW480, and SW620; F-12K - A549 and PC-3. All cell lines were obtained from ATCC or authenticated sources and routinely tested for mycoplasma contamination. Cells were passaged at 70–80% confluence using 0.25% trypsin-EDTA.

#### Mouse models

Female NSG mice were purchased from Jackson Laboratory (Strain#005557). All animal surgeries, husbandry and handling protocols were completed according to University of California IACUC guidelines.

#### I-SPY 2 trial overview

All clinical blood samples were received from the I-SPY 2 trial (NCT01042379), an ongoing, open-label, randomized, multicenter adaptive, phase 2 platform trial. Detailed description of the study design, patient eligibility and enrollment and oversight of the trial have been published previously.^49^^,^^50^ The protocol for the I-SPY 2 trial was approved by the Institutional Review Boards at all participating institutions. All patients signed written informed consent to participate in the trial and to allow the use of their biospecimens for research purposes. Blood samples from a cohort of 192 patients were included in this study. Detailed demographic information are reported in Figure S6A.

### Method details

#### Identification of oncRNAs in The Cancer Genome Atlas

11,082 TCGA small RNA-seq data across 32 cancer types and 23 tumor-adjacent normal tissue types were downloaded from the Genomic Data Commons (GDC) in BAM format (hg38). Sample metadata was fetched using the GDC API. Reads were given a sequence complexity score using the DUST algorithm and removed from downstream analysis if the associated sequence complexity fell below a threshold (DUST score >3).^51^ After conversion to BED format, unique small RNA loci across all samples were merged using mergeBed to create a comprehensive list of expressed small RNA loci. Loci longer than 200 base pairs were split via peak calling with SciPy’s (v.1.5) find_peaks function with the following parameters: height = 10, width = [15, 200], distance = 20.

Non-cancerous extracellular and biofluid smRNA-seq data from the exRNA Atlas were downloaded in FASTQ format from the Gene Expression Omnibus (GEO) and the database of Genotypes and Phenotypes (dbGAP) and preprocessed in accordance with the cognate library preparation. Reads were then aligned to the genome (hg38) to generate BAM files. After applying the above low-complexity sequence filter, reads were converted to BED format. IntersectBed was used to create binary TCGA smRNA loci tables to record the presence or absence of the smRNA loci in each exRNA Atlas sample. A smRNA locus is considered present in a sample if at least one smRNA sequence in the sample aligns and overlaps with the relevant locus. SmRNA loci observed in more than 0.43% exRNA Atlas samples were removed. The sample threshold was selected by using an elbow plot.

After filtering the TCGA smRNA loci by exRNA Atlas samples, we used the smRNA loci list to generate counts for each TCGA sample. The resulting smRNA loci counts, library size normalized counts (counts per million), and metadata for each sample were saved in a NoSQL database (MongoDB), aggregated and indexed by the smRNA loci.

To identify “orphan” smRNAs across TCGA, we first applied a filter to retain smRNAs that were largely absent in normal samples. Tumor-adjacent normal samples from TCGA were first stratified based on tissue type. SmRNAs that were observed in more than 10% of normal samples for any of the tissue types were removed. Only tissues with at least 10 normal samples were used for this normal tissue filtering step, which included 14 different tissue types. We then removed RNAs that were largely absent in cancer samples. For this step, we stratified cancer samples into 32 tissue types, and only retained smRNAs that were present in at least 10% of the cancer samples for at least one tissue type. For each cancer tissue type, we then used Fisher’s exact test to compare the presence and absence of the remaining smRNAs of tumor samples from the cognate cancer tissue type and normal samples from all tissue types. We selected smRNAs that were significantly present in the tumor samples of at least one tissue type, using an FDR cutoff of 0.1. After discovery of cancer-enriched smRNA loci, we then filtered our list of annotations against known smRNAs and miRNAs from publicly available annotations. SmRNAs overlapping by genomic coordinate with any of the existing annotations were removed. Lastly, we applied a filter using smRNA-seq libraries from 30 non-cancerous serum samples (cell-free RNA sequencing described below). Cancer-enriched smRNA loci that were detected in more than one of the samples were removed. Only smRNAs that pass our low-complexity sequence filter, non-cancerous extracellular and biofluid samples filter, known smRNA filters, presence in a minimum number of cancer samples (10%) requirement, presence in a maximum number of tumor-adjacent normal samples (10%) requirement, and significant association with cancer (Fisher’s exact test) were included in our final annotated list of high-confidence oncRNAs.

#### Cancer tissue-of-origin modeling

To evaluate the utility of oncRNAs for cancer tissue-of-origin modeling, we first divided the TCGA cancer samples into a training cohort and a held-out testing cohort using an 80/20 train/test ratio, stratified by cancer types. We used the same methodology to train our classifier models on binarized, “digital” oncRNA profiles and normalized oncRNA expression profiles. Within the training cohort (80% of all cancer samples), we performed recursive feature elimination in a 5-fold cross validation scheme using a XGBoost classifier as our estimator to reduce the number of oncRNAs used as features from 260,968 to 1,805 (binary) features and 1,805 (cpm) features. After feature selection, we trained a final XGBoost classifier with 500 trees at max-depth of 3 on the full training cohort. The final model was evaluated on the held-out test set (20% of all cancer samples) to calculate accuracy, precision, recall, and f1-scores.

#### oncRNA validation in the Clinical Proteomic Tumor Analysis Consortium data

938 CPTAC small RNA-seq data from tumors across 6 cancer types (breast carcinoma, colorectal cancer, lung adenocarcinoma, lung squamous cell carcinoma, pancreatic ductal adenocarcinoma, and uterine corpus endometrial carcinoma) were downloaded from GDC in BAM format (hg38). Corresponding clinical data was also downloaded from GDC. Count tables of oncRNAs were generated from the sequencing data using bedtools (Intersectbed) and our annotated list of high-confidence oncRNAs. Detailed distribution of cancer samples used in this study are listed in Table S4.

To confirm the reproducibility of oncRNA-based digital barcodes for cancer identity, we used TCGA cancer samples of matching cancer types in the CPTAC data to train a model for cancer tissue-of-origin prediction. Within the TCGA training cohort (*N* = 3,446), we first performed recursive feature elimination in a 5-fold cross validation scheme using a XGBoost classifier as our estimator to reduce the number of oncRNAs used as features from all oncRNAs observed in the TCGA training cohort to 2,313 oncRNAm (binary) features. After feature selection, we trained a final XGBoost classifier with 300 trees at max-depth of 3 on the full TCGA training cohort. The final model was evaluated on the held-out CPTAC cancer samples to calculate accuracy, precision, recall, and f1-scores.

#### oncRNA and chromatin accessibility association analysis

TCGA chromatin accessibility data were downloaded from GDC Publication Page (https://gdc.cancer.gov/about-data/publications/ATACseq-AWG). Of the 404 unique donors in the published study, 386 had matching TCGA smRNAseq data and were selected for inclusion in the analysis. Raw count matrices of published pan-cancer peaks of chromatin accessibility were normalized by library size. We then used intersectBed to identify ATAC peaks that overlapped with our set of oncRNA loci. To search for novel transcriptional activity, we removed any oncRNAs that overlapped with known genomic annotations, resulting in 10,725 oncRNA-ATAC peak pairs. For oncRNA-ATAC peak overlaps with at least 5 samples expressing the corresponding oncRNA, we performed a one-tailed Mann Whitney U test to test for higher ATAC peak scores in samples that expressed the cognate oncRNA compared to samples in which the oncRNA was not detected. *p* values were FDR corrected, resulting in 1,989 significant associations.

#### Cancer subtype analysis and modeling

Clinical metadata with subtype information for TCGA-BRCA datasets and TCGA-CRC (COAD and READ) were downloaded from cBioPortal (https://www.cbioportal.org/) and the Sage Bionetworks Synapse (https://www.synapse.org/), respectively. For each cancer, we used all oncRNAs found to be statistically enriched in the cancer to train and evaluate XGBoost classifiers to predict cancer subtypes (Basal, Her2, Luminal A, and Luminal B for BRCA; CMS1, CMS2, CMS3, and CMS4 for CRC) in a 5-fold cross-validation setup. For both BRCA and CRC we used XGBoost classifiers with 100 trees at max-depth of 3. During cross-validation, the samples are first divided into 5 groups, each consisting of 20% of the samples to serve as the held-out set for performance evaluation for each fold. For each of the 5 held-out groups, we used remaining samples not in the held-out group (80% of the data) for model training and the held-out group (20% of the data) for assessing the performance metrics of the model within the fold. Performance metrics of the models including AUC of ROC, precision, recall, f1-score, and accuracy were averaged across folds.

#### oncRNA selection for functional screens

We triaged our list of ∼260,000 of oncRNAs to select target oncRNAs for inclusion in our *in vivo* over-expression and loss-of-function screens. oncRNAs were prioritized based on higher expression levels and prevalence across different cell line models of breast (MDA-MB231), colon (SW480), lung (A549), and prostate (C4-2B) cancers. Selected oncRNA loci longer than 38nt were trimmed to capture the region with the highest coverage or split into multiple smaller target loci if uniform coverage across the cell lines. The lengths of candidate oncRNA loci ranged from 15 to 38 nt after trimming for optimal performance in our TuD constructs.

#### Library cloning

For our combined oncTuD library, a library of 788 oligos (consisting of nominated oncRNAs as well as their corresponding TuD constructs) was designed and ordered from Twist Biosciences. The pool was resuspended to 5ng/uL final concentration in Tris-HCl 10mM pH 8, and a qPCR to determine Ct to be used for downstream library amplification was performed (forward primer: ATTTTGCCCCTGGTTCTT, reverse primer: CCCTAAGAAATGAACTGG) using a 16-fold library dilution.

#### TuD library

For TuDs, the library was then amplified via PCR and ran out on a 2% agarose gel to check library size (expected band of 200bp). PCR product was then cleaned up using a DNA Clean and Concentrator kit-5 (Zymo Research Cat. #D4003), and eluted in 25 μL H_2_O. Cleaned product was digested for 90 min using FD Esp3I (Thermo Fisher Cat. #FD0454). Digested inserts were run on a 8% TBE gel and extracted, and ethanol precipitated overnight in −20C. Inserts were then ligated into pUC6 (Addgene plasmid #49793) in a 100ng reaction with 1:1 insert:backbone ratio for 16h 16C. Ligated products were then ethanol precipitated overnight at −20C, and eluted in 4.5 μl H_2_O. 1.5 μl ligation product was used for electroporation into 20 μl MegaX DH10B T1^R^ electrocompetent cells (Invitrogen Cat. #C640003), followed by maxiprep plasmid isolation.

5 μg of intermediate pUC6 ligation product was then digested for 90 min using AgeI-HF (New England Biolabs Cat. #R3552S) and EcoRI-HF (New England Biolabs Cat. #R3101S). Digested inserts were then run on a 8% TBE gel, extracted, and then ethanol precipitated overnight at −20C. Inserts were then ligated into pLKO.1 (Addgene plasmid #10878) in a 100ng reaction with 1:1 insert:backbone ratio for 16 h at 16C. Ligated products were then ethanol precipitated overnight at −20C, and eluted in 4.5 μl H_2_O. 1.5 μl ligation product was used for electroporation into 20 μl MegaX DH10B T1^R^ electrocompetent cells (Invitrogen Cat. #C640003), followed by maxiprep plasmid isolation.

#### oncRNA library

For oncRNAs, the library was then amplified via PCR and ran out on a 2% agarose gel to check library size (expected band of 75bp). PCR product was then cleaned up using a DNA Clean and Concentrator kit-5 (Zymo Research Cat. #D4003), and eluted in 25 μL H_2_O. Cleaned product was digested for 90 min using AgeI-HF (New England Biolabs Cat. #R3552S) and EcoRI-HF (New England Biolabs Cat. #R3101S). Digested inserts were ran on a 8% TBE gel and extracted, and ethanol precipitated overnight in −20C. Inserts were then ligated into pLKO.1 (Addgene plasmid #10878) in a 100ng reaction with 1:1 insert:backbone ratio for 16 h at 16C. Ligated products were then ethanol precipitated overnight at −20C, and eluted in 4.5 μl H_2_O. 1.5 μl ligation product was used for electroporation into 20 μl MegaX DH10B T1^R^ electrocompetent cells (Invitrogen Cat. #C640003), followed by maxiprep plasmid isolation.

#### Sequencing validation

For sequencing validation, 300ng plasmid DNA was used as input to a first PCR targeting the oncTuD amplicon (forward primer: GGAAAGGACGAAACACCGGT; reverse primer: ATACTGCCATTTGTCTCGAGGTC) in 50 μl volume, and PCR product was cleaned up using a Qiagen MinElute PCR purification kit, using a 1:1 volume of NTI cleanup buffer and eluting in 10 μl volume (Qiagen Cat. #28004). 2 μl of PCR product was then used as input into a second PCR to add Illumina adapter sequences (forward primer: ACACTCTTTCCCTACACGACGCTCTTCCGATCTGGAAAGGACGAAACACCGGT; reverse primer: GTGACTGGAGTTCAGACGTGTGCTCTTCCGATCTATACTGCCATTTGTCTCGAGGTC) in 50 μl volume, and PCR product was cleaned up using Qiagen MinElute PCR purification kit with 1:4 NTI and eluting in 10 μl volume. All 10 μl of PCR product from the previous PCR was used as input into a final third indexing PCR to add Illumina indices (Illumina TruSeq UDI indices UDI009-0017). PCR product was cleaned up using 1× left-hand size selection (Zymo Cat. #D4084-4-10). Samples were then pooled and sequenced using a MiSeq v2 kit (Illumina Cat. #MS-102-2002).

#### Lentivirus titration

2×10^5^ cells per cell line (MDA-MB-231, SW480, C4-2B, A549) were seeded into 6-well plates (day 0). 24 h post-seeding (day 1), 2 wells were counted and cell number per cell line recorded. To calculate titer, lentiviral library was added in an upwards range (100, 250, 500 μl) in 3 wells per cell line. 72 h post-seeding (day 3), puromycin was added to transduced wells, as well as an untransduced ‘kill’ well, at 8 μg/mL final concentration. 3 days post-transduction (day 6), all wells were counted, as well as 2 untransduced and non-selected wells. Based on recorded cell number, one selected well per cell line (targeting 10–30% MOI) was used moving forward and expanded for future experiments.

#### Cell preparation for subcutaneous injection

Transduced cells were partitioned into 3 arms for our *in vivo* functional oncTuD screen. 2×10^5^ cells per cell line were split into a 15cm plate for *in vitro* long-term passage, for purposes of growth normalization. 2×10^5^ cells per cell line were also pelleted and frozen at −80C for downstream t0 gDNA extraction. For MDA cells, 16 million cells were resuspended to final concentration 1×10^6^ cells/50μl in 1:1 PBS/matrigel, and bilateral mammary fat pad injections in 50 μl final volume were performed in female, 8-12 week-old age-matched female NOD *scid* gamma (NSG) mice (*n* = 4). For SW480, C4-2B, and A549 cells, 16 million cells per cell line were resuspended to final concentration 1×10^6^ cells/200μl in 1:4 PBS/matrigel, and bilateral subcutaneous injections in 200 μl final volume were performed in either male (C4-2B) or female (SW480, A549) 8-12 week-old age-matched NSG mice (*n* = 4 per cell line).

#### Tumor gDNA extraction and library preparation

3-4 weeks post-injection, tumors were harvested and processed using Quick-DNA midiprep plus kit (Zymo Research Cat. #D4075). For each processed tumor, gDNA was amplified in the ratio of 2.5 μl input/25μl reaction volume in a first PCR targeting the oncTuD amplicon (forward primer: GGAAAGGACGAAACACCGGT; reverse primer: ATACTGCCATTTGTCTCGAGGTC). PCR product was cleaned up using 1× left-hand size selection (Zymo Cat. #D4084-4-10). 10% input from the first PCR was used in a second PCR to add Illumina adapter sequences (forward primer: ACACTCTTTCCCTACACGACGCTCTTCCGATCTGGAAAGGACGAAACACCGGT; reverse primer: GTGACTGGAGTTCAGACGTGTGCTCTTCCGATCTATACTGCCATTTGTCTCGAGGTC), and PCR product was cleaned up using 1× left-hand size selection (Zymo Cat. #D4084-4-10). 10% input from the second PCR was used in a last indexing PCR to add Illumina indices (Illumina TruSeq UDI indices UDI001-080), followed by 1× left-hand size selection (Zymo Cat. #D4084-4-10). Samples were pooled and sequenced on 2 lanes of NovaSeq SP200 150x8x8×50 at the UCSF Center for Advanced Technology (CAT).

#### Immunofluorescence imaging

2.5×10^5^ cells were seeded per well of a 6-well plate in culture medium. 72 h later, the cell culture medium was removed and wells were rinsed 3 times using PBS-T. Cells were fixed using 5% paraformaldehyde (pH 7.4) for 15 min at room temperature then washed three times with PBS-T. 0.1% Triton X-100 in PBS was used to permeabilize the cells for 5 min at room temperature. After three additional washes, 10% goat serum (Thermo Fisher #50197Z) in PBS was added and the cells were incubated at room temperature for 60 min 1:100 dilution of rabbit anti-vimentin antibody (Abcam #ab92547) in 1% goat serum was added to the cells and incubated overnight at 4°C. For the control, a 1:500 dilution of human IgG Fc isotype control (Abcam #ab206214) in 1% goat serum was used under identical conditions. After washing, 2 μg/mL of goat anti-rabbit secondary antibody (Thermo Fisher #A-11034) in 0.1% goat serum was added and incubated for 45 min at room temperature protected from light. Samples were washed and DAPI nuclear stain was added. After final washes, cells were imaged using an Echo Revolve microscope. Images were analyzed using CellProfiler to detect cells and measure vimentin signal intensity (v4.2.8).^52^

#### Orthotopic tumor growth assay

Tumor growth assays were performed by injecting cancer cells (5×10^5^ MDA-MB-231 or HCC1806-LM2 shctrl, oncRNA.ch7.29, or oncRNA.ch17.67) in 50μL 1:1 PBS:Matrigel (Corning) bilaterally into mammary fat pads of eight-to twelve-week old age-matched female NOD/SCID gamma mice. Tumor volume was assessed weekly by caliper measurements. Final tumor volume was measured *ex vivo* after surgically removing the tumor.

#### Metastatic lung colonization assay

Eight-to twelve-week-old age-matched female NOD/SCID gamma mice (NSG, Jackson Labs, 005557) were used for lung colonization assays. For this assay, cancer cells constitutively expressing luciferase were suspended in 100 μL PBS and then injected via tail-vein (1×10^5^ MDA-MB-231 or HCC1806-LM2 shctrl, oncRNA.ch7.29, or oncRNA.ch17.67). Each cohort contained 4–5 mice, which in the NSG background is enough to observe a >2--fold difference with 90% confidence. Mice were randomly assigned into cohorts. Cancer cell growth was monitored *in vivo* at the indicated times by retro-orbital injection of 100 μL of 15 mg/mL luciferin (PerkinElmer) dissolved in 1× PBS, and then measuring the resulting bioluminescence with an IVIS instrument and Living Image software (PerkinElmer).

#### Cell line mRNA sequencing

RNA was extracted extracted using the Zymo Research Quick-RNA Microprep Kit (cat#R1051) in replicates from MDA-MB-231 or HCC1806-LM2 shctrl, oncRNA.ch7.29, or oncRNA.ch17.67; 100-200ng RNA was then used as input to QuantSeq FWD. mRNA-seq libraries were constructed using the QuantSeq 3′ mRNA-Seq Library Prep Kit FWD according to the manufacturer’s instructions (Lexogen, Cat. #015). mRNA-seq libraries were pooled and sequenced on 1 lane of NovaSeqX 100x6x0×0 at the UCSF Center for Advanced Technology (CAT).

#### Conditioned media collection and cell-free smRNA sequencing

For each cancer of the 25 cancer lines, 200k-300k cells were seeded into a well of a 6-well plate in biological duplicate. After 48 h, media was aspirated, cells were washed with PBS, then 3mL of fresh media prepared with exosome-depleted FBS was added. After 24 h, conditioned media was collected, then cell-free RNA was extracted immediately with Quick-cfRNA Serum and Plasma kit (Zymo) and flash frozen. CfRNA was quantified with Qubit RNA HS, and ∼14ng of each sample was used as input to construct small RNA-seq libraries with SMARTer smRNA-Seq Kit (Takara). For library prep, two modifications were made from the manufacturer’s protocol: (a) the stock oligo dT for first strand synthesis was substituted for a custom primer with UMI’s (5′CAAGCAGAAGACGGCATA.

CGAGATNNNNNNNNGTGACTGGAGTTCAGACGTGTGCTCTTCCGATCTTTTTTTTTTTTTTT-3′) and (b) custom primers with single i5 indices were used for 18 cycles of cDNA amplification. For cleanup, the PCR products were column purified as per manufacturer’s recommendations, and 175–300 bp PCR products were gel-purified from 8% polyacrylamide gels in TBE buffer. When necessary, the resulting libraries were additionally PCR-amplified with universal primers (5′-AATGATACGGCGACCACC-3′ and 5′-CAAGCAGAAGACGGCATACGAG-3′). The libraries were sequenced on Illumina HiSeq 4000 or NovaSeq machines at the UCSF Center for Advanced Technology, on double-indexed single-end 50 nt runs.

#### I-SPY 2 trial clinical samples

Blood samples from the I-SPY 2 trial were collected at pretreatment (T0), and after NAC before surgery (T3) in marble/tiger-top vacutainer (serum separator) tubes. Tubes were placed upright for at least 15 min to properly clot. Within two hours of collection, tubes were centrifuged at 2500 rpm for 20 min at room temperature and then aliquoted into cryovial tubes and immediately frozen at −80C for storage.

#### Serum RNA extraction and sequencing

For cell-free RNA extraction from patient serum samples, 0.5–1 mL of serum (stored at −80C from collection to extraction) per sample was used. The samples were thawed at room temperature and RNA was extracted using Quick-cfRNA Serum and Plasma kit (Zymo) following manufacturer’s recommendations, eluted in 15 μL nuclease-free water and stored at −80C. Small RNA-seq libraries were constructed, sequenced and analyzed as described above for cell line conditioned media cell-free RNA.

### Quantification and statistical analysis

All software used was described in the main text or the appropriate methods section. Statistical tests, as well as statistical comparisons between groups, for each figure were denoted in the corresponding figure legend. *p* values for each statistical test were noted in each figure panel or relevant text. Threshold of significance for (adjusted) *p* values was set at 0.05 unless otherwise stated.

#### Analysis of oncRNA and oncTuD functional screen results

Sequencing results from the functional screen were processed using cutadapt (v. 3.5) to remove adapter sequences and aligned to our selected oncRNA reference using bowtie2 (v.2.3.5.1). We used DESeq2 (v. 1.26.0) with default settings to compare normalized counts between *in vivo* grown tumors and *in vitro* controls.^53^ *p* values were FDR corrected with a significance threshold of adjusted *p* < 0.05.

#### Target oncRNA expression and clinical association in TCGA-BRCA

For oncRNAs with potential functional roles, we used the associated TCGA clinical metadata to compare their expression across tumor-adjacent normal tissue and cancer tissue and across breast cancer subtypes. We also stratified patients based on the expression levels of the oncRNAs and generated Kaplan-Meier curves. A log rank test was used to compare the resulting survival curves.

#### TCGA differential expression analysis and pathway analysis

Raw gene expression data for the TCGA-BRCA dataset were downloaded from the Genomic Data Commons. Expression data were processed and normalized following the guidelines of the edgeR pipeline. Samples were grouped by presence or absence of cognate oncRNA and compared for differentially expressed genes using edgeR (v. 3.42.4), controlling for covariates including age and breast cancer subtype.^54^ The resulting *p* values and log-fold change of each gene were used by iPage for pathway analysis to identify pathway perturbations associated with oncRNA expression.^27^

#### Analysis of cell line mRNA sequencing

We used cutadapt (v. 3.5) to remove adapter sequences. Preprocessed sequences were pseudoaligned to the transcriptome with Salmon (v. 0.14.1) to quantify gene expression. We used DESeq2 (v. 1.26.0) to perform the differential expression analysis with default settings.^53^ *p* values were FDR corrected and used with gene expression data for pathway analysis with iPage, as mentioned above.

#### Analysis of cell-free smRNA sequencing

We used cutadapt (v1.15) to remove the poly(A) tails from the 3′ end and 3 nucleotides unconditionally from the 5′ end of each read to remove the template switch oligo. Reads with at least 15 base pairs after trimming were aligned to the human genome (hg38) using bowtie2 (v.2.3.5.1) with the end-to-end and sensitive setting. Libraries with UMIs were deduplicated using UMI-tools (v.1.1.0) with the default directional algorithm setting. The aligned BAM files were converted to BED format and intersectBed was used to quantify the number of reads mapping to known smRNAs (i.e.,: miRNA, tRNA) and our list of annotated oncRNAs.

#### I-SPY 2 survival analysis

Residual oncRNA burden (ΔoncRNA) for each patient was calculated as

$$\Delta \text{oncRNA}=N_{T3}-N_{T0}$$

where N_T0_ and N_T3_ were the total number of oncRNA species detected per million reads sequenced from the serum samples at time point 0 (prior to neoadjuvant chemotherapy) and time point 3 (completion of neoadjuvant chemotherapy treatment and prior to surgery), respectively. Patients were stratified by ΔoncRNA levels into two groups: i) high and persistent residual oncRNA burden and ii) low residual oncRNA burden (Figure S4F). Using these stratifications we generated Kaplein-Meier curves and performed a log rank test to calculate the associated *p* value. We used multivariable Cox regression analysis to assess ΔoncRNA as an independent predictor of survival after NAC while controlling for established clinical variables. To account for the sample size, we performed several iterations of Cox analysis with different covariates separately: ΔoncRNA with pCR, ΔoncRNA with RCB class, and ΔoncRNA with age and breast cancer subtype.

### Additional resources

The I-SPY 2 trial is registered with ClinicalTrials.gov: NCT01042379. Additional information about the I-SPY2 trial can be found in the following links: https://clinicaltrials.gov/study/NCT01042379 and https://www.quantumleaphealth.org/for-patients/i-spy-trials/i-spy2-pre/.

## Supplemental information

Document S1. Figures S1–S6 and Tables S1–S5

Table S6. Raw and adjusted *p* values from oncRNA and oncTuD functional screens, related to Figure 3 (ST6A) Differential expression results for BRCA oncRNA screen. ST6B) Differential expression results for COAD oncRNA screen. ST6C) Differential expression results for LUAD oncRNA screen. ST6D) Differential expression results for PRAD oncRNA screen. ST6E) Differential expression results for BRCA oncTuD screen. ST6F) Differential expression results for COAD oncTuD screen. ST6G) Differential expression results for LUAD oncTuD screen. ST6H) Differential expression results for PRAD oncTuD screen.

Document S2. Article plus supplemental information

## References

[1] Knezevich S.R., McFadden D.E., Tao W., Lim J.F., Sorensen P.H. (1998). A novel ETV6-NTRK3 gene fusion in congenital fibrosarcoma. Nat. Genet..

[2] Larson R.A., Kondo K., Vardiman J.W., Butler A.E., Golomb H.M., Rowley J.D. (1984). Evidence for a 15;17 translocation in every patient with acute promyelocytic leukemia. Am. J. Med..

[3] Rowley J.D. (1973). Letter: A new consistent chromosomal abnormality in chronic myelogenous leukaemia identified by quinacrine fluorescence and Giemsa staining. Nature.

[4] Xie N., Shen G., Gao W., Huang Z., Huang C., Fu L. (2023). Neoantigens: promising targets for cancer therapy. Signal Transduct. Target. Ther..

[5] Smith C.C., Selitsky S.R., Chai S., Armistead P.M., Vincent B.G., Serody J.S. (2019). Alternative tumour-specific antigens. Nat. Rev. Cancer.

[6] Turner K.M., Deshpande V., Beyter D., Koga T., Rusert J., Lee C., Li B., Arden K., Ren B., Nathanson D.A. (2017). Extrachromosomal oncogene amplification drives tumour evolution and genetic heterogeneity. Nature.

[7] Kim H., Nguyen N.P., Turner K., Wu S., Gujar A.D., Luebeck J., Liu J., Deshpande V., Rajkumar U., Namburi S. (2020). Extrachromosomal DNA is associated with oncogene amplification and poor outcome across multiple cancers. Nat. Genet..

[8] Fish L., Zhang S., Yu J.X., Culbertson B., Zhou A.Y., Goga A., Goodarzi H. (2018). Cancer cells exploit an orphan RNA to drive metastatic progression. Nat. Med..

[9] Panet F., Papakonstantinou A., Borrell M., Vivancos J., Vivancos A., Oliveira M. (2024). Use of ctDNA in early breast cancer: analytical validity and clinical potential. npj Breast Cancer.

[10] Chu A., Robertson G., Brooks D., Mungall A.J., Birol I., Coope R., Ma Y., Jones S., Marra M.A. (2016). Large-scale profiling of microRNAs for The Cancer Genome Atlas. Nucleic Acids Res..

[11] Ainsztein A.M., Brooks P.J., Dugan V.G., Ganguly A., Guo M., Howcroft T.K., Kelley C.A., Kuo L.S., Labosky P.A., Lenzi R. (2015). The NIH Extracellular RNA Communication Consortium. J. Extracell. Vesicles.

[12] Li Y., Kang K., Krahn J.M., Croutwater N., Lee K., Umbach D.M., Li L. (2017). A comprehensive genomic pan-cancer classification using The Cancer Genome Atlas gene expression data. BMC Genom..

[13] Lyu B., Haque A. (2018). Deep Learning Based Tumor Type Classification Using Gene Expression Data. bioRxiv.

[14] Guinney J., Dienstmann R., Wang X., de Reyniès A., Schlicker A., Soneson C., Marisa L., Roepman P., Nyamundanda G., Angelino P. (2015). The consensus molecular subtypes of colorectal cancer. Nat. Med..

[15] Hoadley K.A., Yau C., Hinoue T., Wolf D.M., Lazar A.J., Drill E., Shen R., Taylor A.M., Cherniack A.D., Thorsson V. (2018). Cell-of-Origin Patterns Dominate the Molecular Classification of 10,000 Tumors from 33 Types of Cancer. Cell.

[16] Campbell J.D., Yau C., Bowlby R., Liu Y., Brennan K., Fan H., Taylor A.M., Wang C., Walter V., Akbani R. (2018). Genomic, Pathway Network, and Immunologic Features Distinguishing Squamous Carcinomas. Cell Rep..

[17] Li Y., Dou Y., Da Veiga Leprevost F., Geffen Y., Calinawan A.P., Aguet F., Akiyama Y., Anand S., Birger C., Cao S. (2023). Proteogenomic data and resources for pan-cancer analysis. Cancer Cell.

[18] Corces M.R., Granja J.M., Shams S., Louie B.H., Seoane J.A., Zhou W., Silva T.C., Groeneveld C., Wong C.K., Cho S.W. (2018). The chromatin accessibility landscape of primary human cancers. Science.

[19] Chen S., Markett D., Karimzadeh M., Luo Y., Khoroshkin M., Boyraz B., Lee S., Carpenter C., Nguyen P., Garcia K. (2025). Discovery of a tRNA-regulatory transcription factor that suppresses breast cancer metastasis. bioRxiv.

[20] Culbertson B., Garcia K., Markett D., Asgharian H., Chen L., Fish L., Navickas A., Yu J., Woo B., Nanda A.S. (2023). A sense-antisense RNA interaction promotes breast cancer metastasis via regulation of NQO1 expression. Nat. Cancer.

[21] Parker J.S., Mullins M., Cheang M.C.U., Leung S., Voduc D., Vickery T., Davies S., Fauron C., He X., Hu Z. (2009). Supervised risk predictor of breast cancer based on intrinsic subtypes. J. Clin. Oncol..

[22] Schettini F., Brasó-Maristany F., Kuderer N.M., Prat A. (2022). A perspective on the development and lack of interchangeability of the breast cancer intrinsic subtypes. npj Breast Cancer.

[23] Bak R.O., Hollensen A.K., Primo M.N., Sørensen C.D., Mikkelsen J.G. (2013). Potent microRNA suppression by RNA Pol II-transcribed ‘Tough Decoy’ inhibitors. RNA.

[24] Haraguchi T., Ozaki Y., Iba H. (2009). Vectors expressing efficient RNA decoys achieve the long-term suppression of specific microRNA activity in mammalian cells. Nucleic Acids Res..

[25] Xiao L., Zhang T., Zheng K., Xiao Q., Zhang W., Zhang D., Wu D., He C., Zhou Y., Liu Y. (2023). Knockdown of Secernin 1 inhibit cell invasion and migration by activating the TGF-β/Smad3 pathway in oral squamous cell carcinomas. Sci. Rep..

[26] Du X., Shen X., Dai L., Bi F., Zhang H., Lu C. (2020). PSMD12 promotes breast cancer growth via inhibiting the expression of pro-apoptotic genes. Biochem. Biophys. Res. Commun..

[27] Goodarzi H., Elemento O., Tavazoie S. (2009). Revealing Global Regulatory Perturbations across Human Cancers. Mol. Cell.

[28] Earnest-Noble L.B., Hsu D., Chen S., Asgharian H., Nandan M., Passarelli M.C., Goodarzi H., Tavazoie S.F. (2022). Two isoleucyl tRNAs that decode synonymous codons divergently regulate breast cancer metastatic growth by controlling translation of proliferation-regulating genes. Nat. Cancer.

[29] Garcia-Martin R., Wang G., Brandão B.B., Zanotto T.M., Shah S., Kumar Patel S., Schilling B., Kahn C.R. (2022). MicroRNA sequence codes for small extracellular vesicle release and cellular retention. Nature.

[30] Murillo O.D., Thistlethwaite W., Rozowsky J., Subramanian S.L., Lucero R., Shah N., Jackson A.R., Srinivasan S., Chung A., Laurent C.D. (2019). exRNA Atlas Analysis Reveals Distinct Extracellular RNA Cargo Types and Their Carriers Present across Human Biofluids. Cell.

[31] Dhahbi J.M., Spindler S.R., Atamna H., Boffelli D., Mote P., Martin D.I.K. (2013). 5’-YRNA fragments derived by processing of transcripts from specific YRNA genes and pseudogenes are abundant in human serum and plasma. Physiol. Genomics.

[32] Dhahbi J.M., Spindler S.R., Atamna H., Yamakawa A., Boffelli D., Mote P., Martin D.I.K. (2013). 5’ tRNA halves are present as abundant complexes in serum, concentrated in blood cells, and modulated by aging and calorie restriction. BMC Genom..

[33] Wang H., Yee D. (2019). I-SPY 2: a Neoadjuvant Adaptive Clinical Trial Designed to Improve Outcomes in High-Risk Breast Cancer. Curr. Breast Cancer Rep..

[34] Hooykaas M.J.G., Soppe J.A., De Buhr H.M., Kruse E., Wiertz E.J.H.J., Lebbink R.J. (2018). RNA accessibility impacts potency of Tough Decoy microRNA inhibitors. RNA Biol..

[35] Hanahan D., Weinberg R.A. (2011). Hallmarks of cancer: the next generation. Cell.

[36] Polyak K., Weinberg R.A. (2009). Transitions between epithelial and mesenchymal states: acquisition of malignant and stem cell traits. Nat. Rev. Cancer.

[37] Park S.-M., Gaur A.B., Lengyel E., Peter M.E. (2008). The miR-200 family determines the epithelial phenotype of cancer cells by targeting the E-cadherin repressors ZEB1 and ZEB2. Genes Dev..

[38] Gregory P.A., Bert A.G., Paterson E.L., Barry S.C., Tsykin A., Farshid G., Vadas M.A., Khew-Goodall Y., Goodall G.J. (2008). The miR-200 family and miR-205 regulate epithelial to mesenchymal transition by targeting ZEB1 and SIP1. Nat. Cell Biol..

[39] Kent L.N., Leone G. (2019). The broken cycle: E2F dysfunction in cancer. Nat. Rev. Cancer.

[40] Lynce F., Shajahan-Haq A.N., Swain S.M. (2018). CDK4/6 inhibitors in breast cancer therapy: Current practice and future opportunities. Pharmacol. Ther..

[41] Chen H.-Z., Tsai S.-Y., Leone G. (2009). Emerging roles of E2Fs in cancer: an exit from cell cycle control. Nat. Rev. Cancer.

[42] Magbanua M.J.M., Swigart L.B., Wu H.T., Hirst G.L., Yau C., Wolf D.M., Tin A., Salari R., Shchegrova S., Pawar H. (2021). Circulating tumor DNA in neoadjuvant-treated breast cancer reflects response and survival. Ann. Oncol..

[43] Magbanua M.J.M., Brown Swigart L., Ahmed Z., Sayaman R.W., Renner D., Kalashnikova E., Hirst G.L., Yau C., Wolf D.M., Li W. (2023). Clinical significance and biology of circulating tumor DNA in high-risk early-stage HER2-negative breast cancer receiving neoadjuvant chemotherapy. Cancer Cell.

[44] Schwarzenbach H., Hoon D.S.B., Pantel K. (2011). Cell-free nucleic acids as biomarkers in cancer patients. Nat. Rev. Cancer.

[45] Wang H., Peng R., Wang J., Qin Z., Xue L. (2018). Circulating microRNAs as potential cancer biomarkers: the advantage and disadvantage. Clin. Epigenetics.

[46] Reggiardo R.E., Maroli S.V., Peddu V., Davidson A.E., Hill A., LaMontagne E., Aaraj Y.A., Jain M., Chan S.Y., Kim D.H. (2023). Profiling of repetitive RNA sequences in the blood plasma of patients with cancer. Nat. Biomed. Eng..

[47] Wang J., Huang J., Hu Y., Guo Q., Zhang S., Tian J., Niu Y., Ji L., Xu Y., Tang P. (2024). Terminal modifications independent cell-free RNA sequencing enables sensitive early cancer detection and classification. Nat. Commun..

[48] Larson M.H., Pan W., Kim H.J., Mauntz R.E., Stuart S.M., Pimentel M., Zhou Y., Knudsgaard P., Demas V., Aravanis A.M., Jamshidi A. (2021). A comprehensive characterization of the cell-free transcriptome reveals tissue- and subtype-specific biomarkers for cancer detection. Nat. Commun..

[49] Park J.W., Liu M.C., Yee D., Yau C., van 't Veer L.J., Symmans W.F., Paoloni M., Perlmutter J., Hylton N.M., Hogarth M. (2016). Adaptive Randomization of Neratinib in Early Breast Cancer. N. Engl. J. Med..

[50] Rugo H.S., Olopade O.I., DeMichele A., Yau C., van 't Veer L.J., Buxton M.B., Hogarth M., Hylton N.M., Paoloni M., Perlmutter J. (2016). Adaptive Randomization of Veliparib-Carboplatin Treatment in Breast Cancer. N. Engl. J. Med..

[51] Morgulis A., Gertz E.M., Schäffer A.A., Agarwala R. (2006). A fast and symmetric DUST implementation to mask low-complexity DNA sequences. J. Comput. Biol..

[52] Stirling D.R., Swain-Bowden M.J., Lucas A.M., Carpenter A.E., Cimini B.A., Goodman A. (2021). CellProfiler 4: improvements in speed, utility and usability. BMC Bioinf..

[53] Love M.I., Huber W., Anders S. (2014). Moderated estimation of fold change and dispersion for RNA-seq data with DESeq2. Genome Biol..

[54] Robinson M.D., McCarthy D.J., Smyth G.K. (2010). edgeR: A Bioconductor package for differential expression analysis of digital gene expression data. Bioinformatics.

