# Supplementary material for: Systematic annotation of orphan RNAs reveals blood-accessible molecular barcodes of cancer identity and cancer-emergent oncogenic drivers
Source: Cell Rep Med. 2026 Jan 23;7(2):102577. doi: 10.1016/j.xcrm.2025.102577 (PMC12923976; doi:10.1016/j.xcrm.2025.102577)
Supplement: Document S1. Figures S1–S6 and Tables S1–S5 [file mmc1.pdf]

Cell Reports Medicine, Volume 7

Supplemental information

Systematic annotation of orphan RNAs reveals blood-accessible molecular barcodes of cancer identity and cancer-emergent oncogenic drivers

Jeffrey Wang, Jung Min Suh, Brian J. Woo, Albertas Navickas, Kristle Garcia, Keyi Yin, Lisa Fish, Taylor Cavazos, Benjamin Hänisch, Daniel Markett, Gillian L. Hirst, Lamorna Brown-Swigart, Laura J. Esserman, Laura J. van `t Veer, and Hani Goodarzi

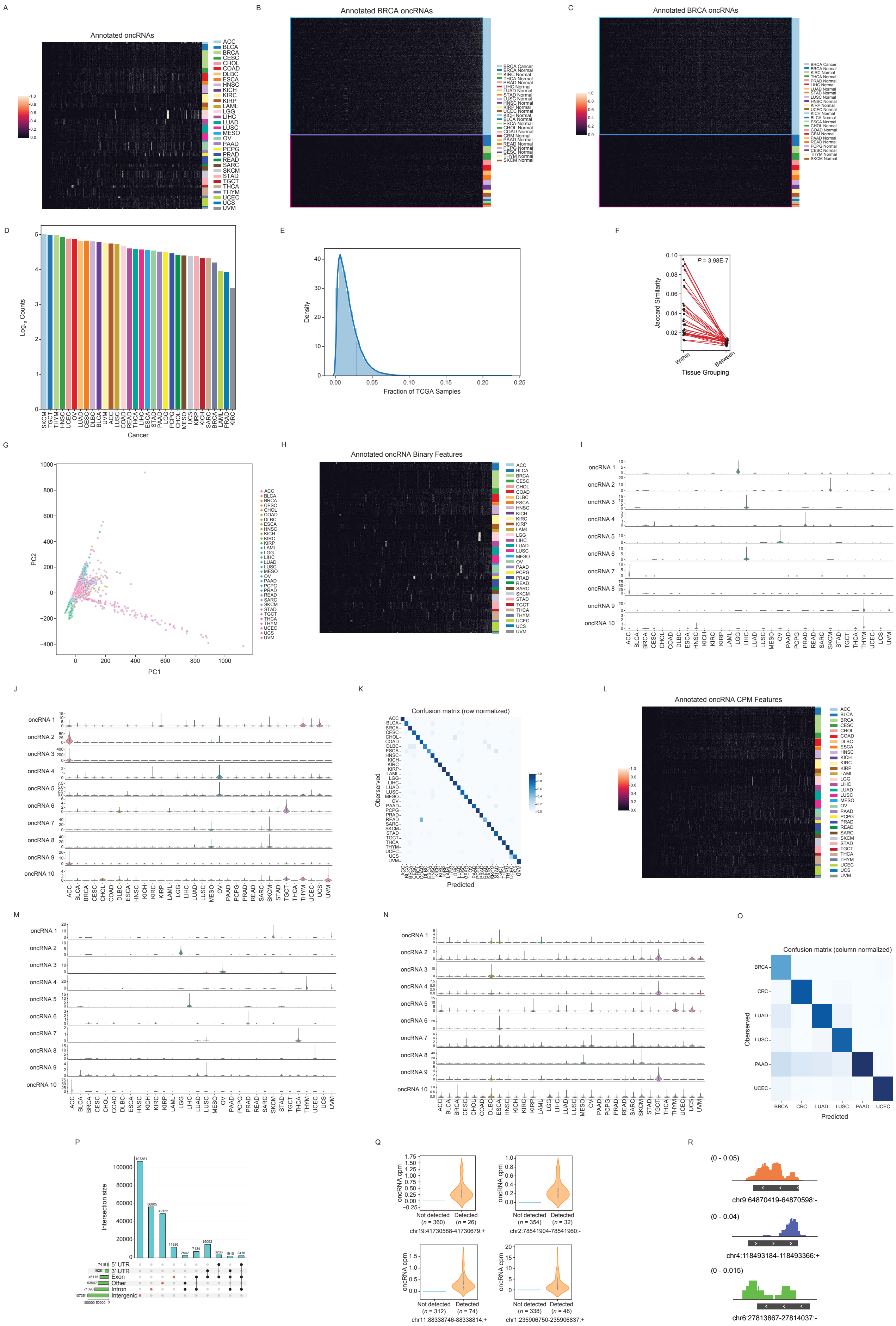

A
Annotated oncRNAs
B
Annotated BRCA oncRNAs
C
Annotated BRCA oncRNAs
D
Log10 Counts
Cancer
E
Density
Fraction of TCGA Samples
F
P = 3.98E-7
Jaccard Similarity
Within
Between
Tissue Grouping
G
PC2
PC1
H
Annotated oncRNA Binary Features
I
J
K
Confusion matrix (row normalized)
Oberserved
Predicted
L
Annotated oncRNA CPM Features
M
N
O
Confusion matrix (column normalized)
Oberserved
Predicted
P
Intersection size
5' UTR
3' UTR
Exon
Other
Intron
Intergenic
Q
oncRNA cpm
Not detected
Detected
chr19:41730588-41730679:+
chr2:78541904-78541960:-
chr11:88338746-88338814:+
chr1:235906750-235906837:+
R
chr9:64870419-64870598:-
chr4:118493184-118493366:+
chr6:27813867-27814037:-

Figure S1. Analysis and profiling of cancer specific oncRNAs across 32 cancer types. Related to Figure 1. **(A)** A heat map of counts-per-million (cpm) normalized expression of oncRNAs across all TCGA cancer samples. Each row represents a sample, and each column represents an oncRNA. Rows were grouped based on tumor type, and columns were clustered based on their presence-absence patterns. For visualization purposes, we max-normalized each column. **(B–C)** Binary (B) and cpm (C) normalized expression heatmap of oncRNAs annotated in the TCGA-BRCA cohort, respectively. A total of 15,827 breast cancer oncRNAs were annotated and plotted here. Each row represents a sample, and each column represents an oncRNA. For the binary heatmap, oncRNA presence shown as light beige and absence as black. For visualization purposes, we max-normalized each column for the cpm expression heatmap. **(D)** Log_{10} number of oncRNAs annotated in each cancer type. **(E)** Density plot of the fraction of TCGA samples for which each of the 260,968 onRNAs was observed. **(F)** Median Jaccard similarity of oncRNA profiles between cancer samples from the same cancer tissue group versus different cancer tissue groups. *P* value was calculated using a one-tailed Wilcoxon test. **(G)** PCA plot of oncRNA profiles of all TCGA cancer samples. Points are colored by the cancer types. **(H)** Binary heatmap of the oncRNAs used as binarized features for the tissue-of-origin (TOO) XGBclassifier model across all TCGA cancer samples. Each row represents a sample, and each column represents an oncRNA; oncRNA presence shown as light beige and absence as black. Rows were grouped based on tumor type, and columns were clustered based on their presence-absence patterns. **(I—J)** Expression levels of top 10 important (I) and prevalent (J) oncRNAs in the TOO XGBclassifier model trained on binary oncRNA profiles. Ranking of oncRNA feature importance is based on average information gain as determined by the model during training. Ranking of oncRNA prevalence is based on the number of samples in which each oncRNA was detected. **(K)** The confusion matrix for TOO classification by XGBoost classifier trained on normalized oncRNA expression data from TCGA training samples and evaluated on held-out TCGA cancer samples. The matrix was row-normalized. **(L)** Heatmap of the cpm normalized expression of oncRNAs used as features for the TOO XGBclassifier model in (K) across all TCGA cancer samples. Each row represents a sample, and each column represents an oncRNA. Rows were grouped based on tumor type, and columns were clustered based on their presence-absence patterns. For visualization purposes, we max-normalized each column. **(M–N)** Expression levels of top 10 important (M) and prevalent (N) oncRNAs in the TOO XGBclassifier model trained on normalized oncRNA expression profiles. The oncRNAs were ranked using the same methods described in (I–J) **(O)** The confusion matrix for TOO classification by XGBoost classifier trained on binarized oncRNA expression data from TCGA samples and evaluated on CPTAC cancer samples. The matrix was column-normalized. **(P)** Upset plot depicting the overlaps of oncRNAs with established genomic features. The “Other” category refers to overlaps of oncRNAs to the opposite strand of the genomic features. oncRNAs with no overlaps with the genomic features were placed in the intergenic category. **(Q)** Normalized expression levels of four exemplary oncRNAs. Expression level of cognate oncRNA was used to split samples into detected and not detected groups for the chromatin accessibility analysis (Figure 1F). Values are shown as violin plots and boxplots. The boxplots show the distribution quartiles, and the whiskers show the quartiles ± IQR (interquartile range). Also reported are the number of samples in which the oncRNAs were detected. **(R)** Normalized GRO-seq tracks from MDA-MB-231 cells in our previously published data showing three exemplary oncRNA loci significantly associated with chromatin accessibility in TCGA tumors from (Figure 1E)[1].

A

BRCA Subtype	Precision	Recall	f1-Score
Basal	0.93 (0.072)	0.97 (0.013)	0.94 (0.044)
Her2	0.79 (0.144)	0.62 (0.148)	0.69 (0.114)
LumA	0.80 (0.016)	0.89 (0.029)	0.84 (0.010)
LumB	0.58 (0.054)	0.42 (0.061)	0.48 (0.037)
Accuracy			0.78 (0.017)
Macro-average	0.77 (0.037)	0.72 (0.039)	0.74 (0.035)
Weighted-average	0.77 (0.019)	0.78 (0.017)	0.77 (0.018)

B

CRC Subtype	Precision	Recall	f1-Score
CMS1	0.69 (0.083)	0.65 (0.149)	0.66 (0.083)
CSM2	0.68 (0.033)	0.79 (0.032)	0.73 (0.030)
CMS3	0.56 (0.124)	0.42 (0.082)	0.48 (0.091)
CMS4	0.53 (0.092)	0.47 (0.088)	0.49 (0.083)
Accuracy			0.63 (0.037)
Macro-average	0.61 (0.044)	0.58 (0.046)	0.59 (0.044)
Weighted-average	0.62 (0.039)	0.63 (0.037)	0.62 (0.039)

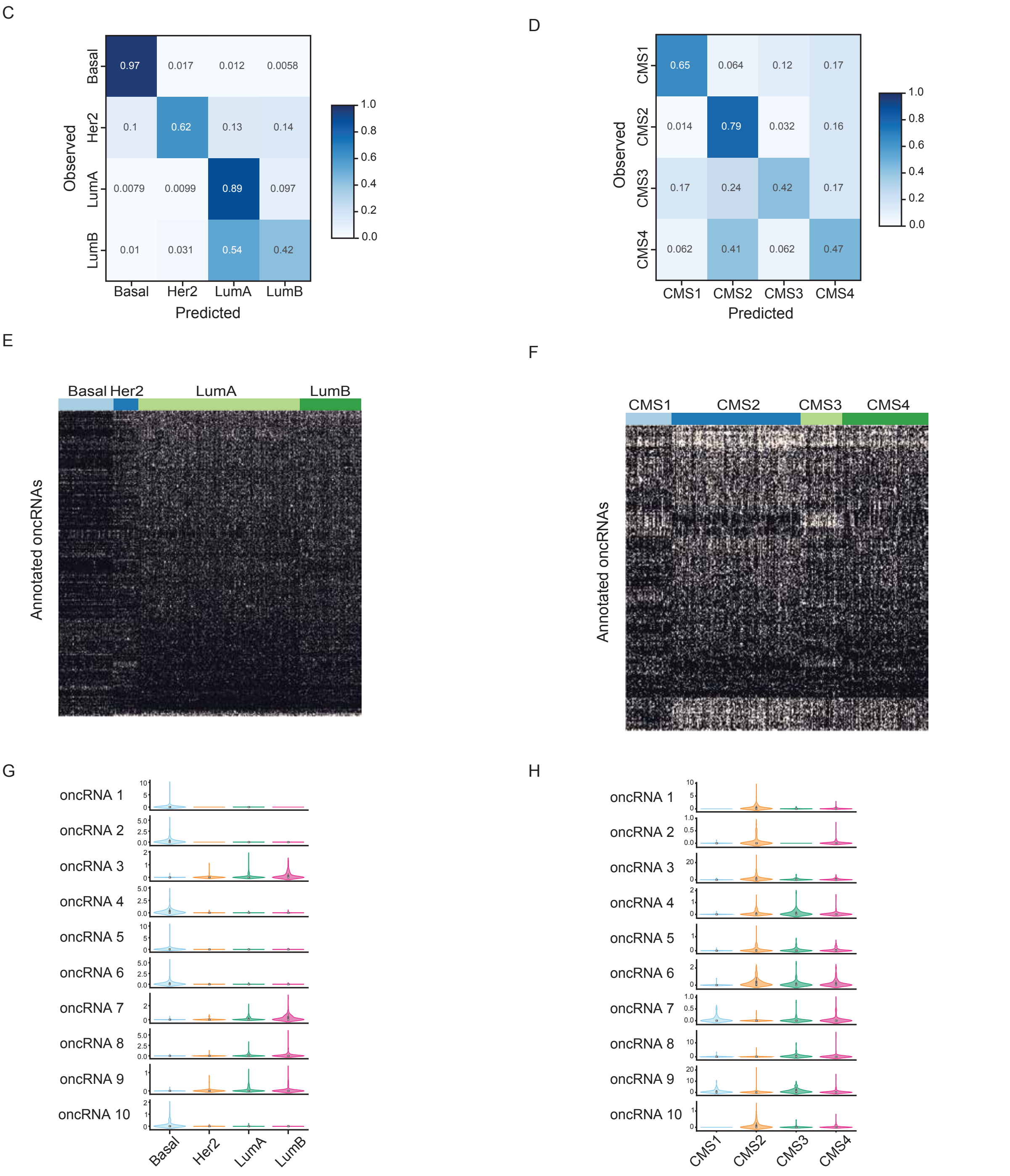

Figure S2. Analysis of subtype specific oncRNAs in breast and colorectal cancers. Related to Figure 2. **(A)** Performance metrics of the breast cancer subtype XGBclassifer averaged (standard deviation) across 5 folds. **(B)** Performance metrics of the colorectal cancer subtype XGBclassifer averaged (standard deviation) across 5 folds. **(C)** The confusion matrix for breast cancer subtype classification averaged across 5 folds for the XGBclassifier. The matrix was row-normalized. **(D)** The confusion matrix for colorectal cancer subtype classification averaged across 5 folds for the XGBclassifier. The matrix was row-normalized. **(E–F)** Binary heatmap of oncRNAs used as features by the XGBclassifier for breast cancer (E) and colorectal cancer (F). Each row represents an oncRNA, and each column represents a sample; oncRNA presence shown as light beige and absence as black. Rows were clusted based on their presence-absence patterns and columns were grouped based on tumor type. **(G–H)** Expression levels of top 10 important oncRNAs in the XGBclassifier models trained on binary oncRNA expression profiles to predict breast cancer subtype (G) and colorectal cancer subtype (H). Ranking of oncRNA feature importance is based on average information gain as determined by the model during training.

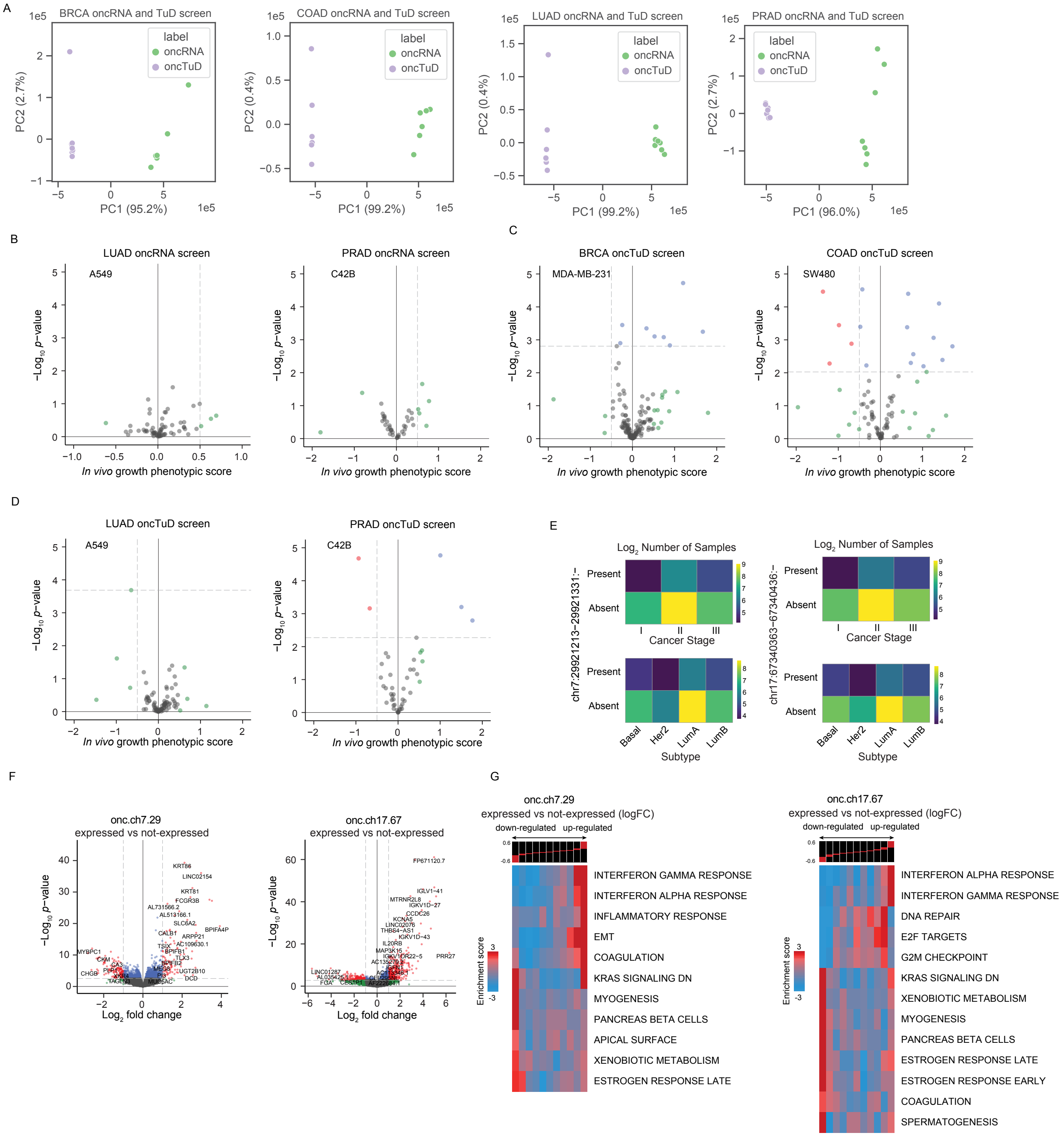
A
BRCA oncRNA and TuD screen
COAD oncRNA and TuD screen
LUAD oncRNA and TuD screen
PRAD oncRNA and TuD screen
label
oncRNA
oncTuD
PC1 (95.2%)
PC2 (2.7%)
PC1 (99.2%)
PC2 (0.4%)
PC1 (96.0%)
1e5
B
LUAD oncRNA screen
A549
PRAD oncRNA screen
C42B
C
BRCA oncTuD screen
MDA-MB-231
COAD oncTuD screen
SW480
In vivo growth phenotypic score
−Log10 p−value
D
LUAD oncTuD screen
A549
PRAD oncTuD screen
C42B
E
Log2 Number of Samples
Present
Absent
Cancer Stage
I
II
III
Basal
Her2
LumA
LumB
Subtype
chr7:29921213−29921331:-
chr17:67340363−67340436:-
F
onc.ch7.29
expressed vs not-expressed
onc.ch17.67
expressed vs not-expressed
Log2 fold change
G
onc.ch7.29
expressed vs not-expressed (logFC)
down-regulated
up-regulated
Enrichment score
INTERFERON GAMMA RESPONSE
INTERFERON ALPHA RESPONSE
INFLAMMATORY RESPONSE
EMT
COAGULATION
KRAS SIGNALING DN
MYOGENESIS
PANCREAS BETA CELLS
APICAL SURFACE
XENOBIOTIC METABOLISM
ESTROGEN RESPONSE LATE
onc.ch17.67
expressed vs not-expressed (logFC)
INTERFERON ALPHA RESPONSE
INTERFERON GAMMA RESPONSE
DNA REPAIR
E2F TARGETS
G2M CHECKPOINT
KRAS SIGNALING DN
XENOBIOTIC METABOLISM
MYOGENESIS
PANCREAS BETA CELLS
ESTROGEN RESPONSE LATE
ESTROGEN RESPONSE EARLY
COAGULATION
SPERMATOGENESIS

Figure S3. *In vivo* screen to identify oncRNAs with functional roles during cancer progression. Related to Figure 3.

(A) PCA plot of oncRNA and oncRNA Tough Decoy (oncTuD) expression in breast (BRCA; MDA-MB-231), colorectal (CRC; SW480), lung (LUAD; A549), and prostate (PRAD; C4-2B) cancer cell lines transduced with cognate oncRNA (green) or oncTuD (purple) libraries. Each cancer gain-of-function and loss-of-function screen was done in replicates. **(B)** Volcano plots of onRNA functional screen results for lung cancer (A549) and prostate cancer (C42B), respectively. *In vivo* growth phenotypic score refers to the representation of cancer cells transduced with cognate oncRNA upon tumor growth in the xenograft model compared to the corresponding *in vivo* baseline. DESeq2 was used to compare *in vivo* and *in vitro* representation and calculate *P* values. Significance threshold was set at adjusted *P* value < 0.05. Significance threshold was set at adjusted *P* value < 0.05. **(C–D)** Volcano plots of onRNA TuD functional screen results for breast cancer (MDA-MB-231) and colorectal cancer (SW480) (C) and lung cancer (A549) and prostate cancer (C42B) (D). *In vivo* growth phenotypic score and *P* values were calculated as described in (B). Significance threshold was set at adjusted *P* value < 0.05. **(E)** Log_2 count (see scale) matrices of TCGA breast cancer samples stratified by cancer stage (top) or subtype (bottom) for which two driver oncRNAs with significant tumor growth phenotype were present or absent. **(F)** Volcano plots of differentially expressed genes in TCGA-BRCA tumors expressing the specified oncRNA compared with tumors in which cognate oncRNA was undetected. The *P* values were calculated using edgeR. Significance threshold was set at adjusted *P* value < 0.05. **(G)** Full list of informative iPage pathways associated with TCGA-BRCA tumors expressing cognate oncRNAs compared to TCGA-BRCA tumors in which respective oncRNAs were not detected.

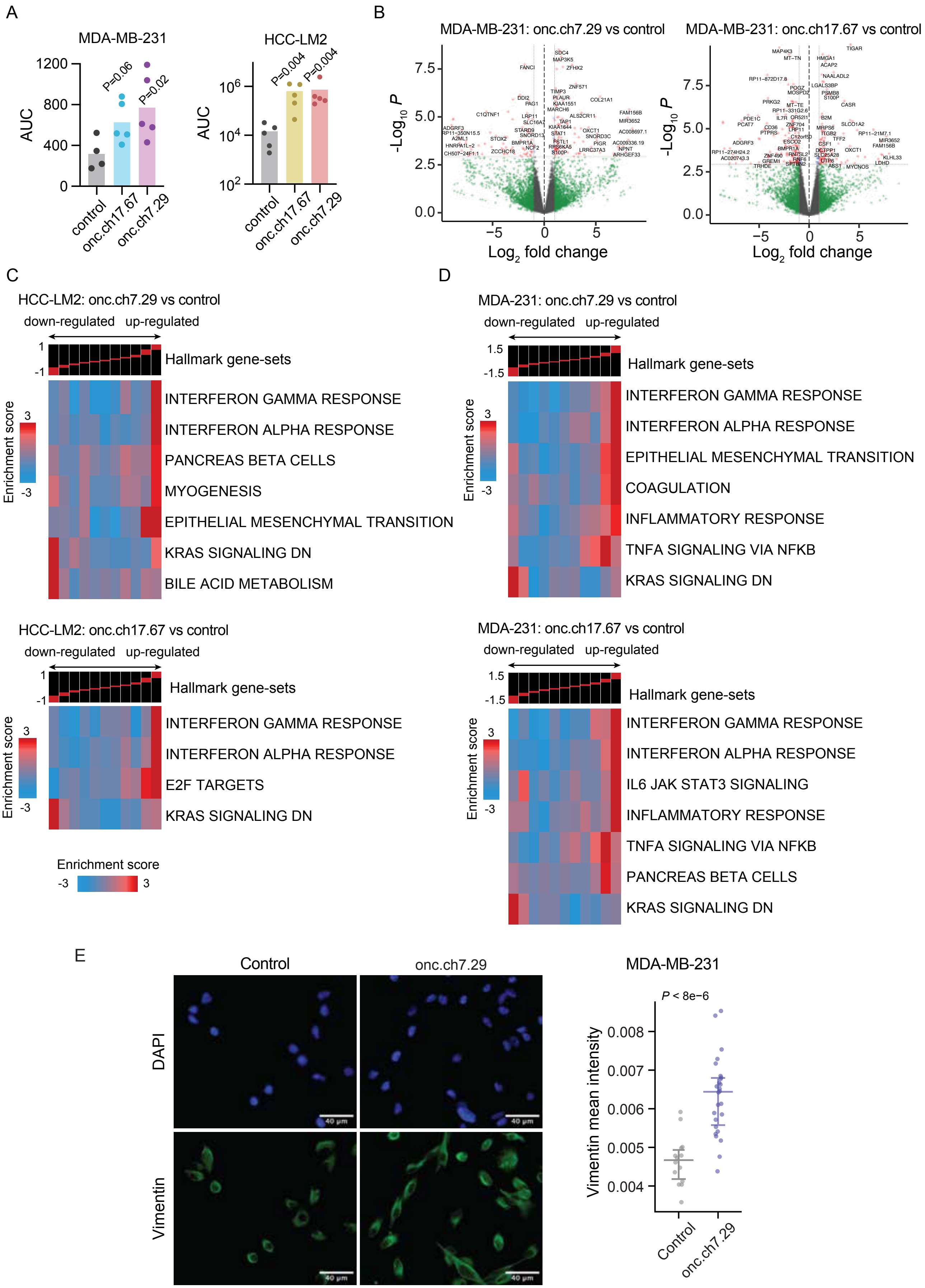

A
MDA-MB-231
HCC-LM2
AUC
P=0.06
P=0.02
P=0.004
P=0.004
control
onc.ch17.67
onc.ch7.29
B
MDA-MB-231: onc.ch7.29 vs control
MDA-MB-231: onc.ch17.67 vs control
-Log10 P
Log2 fold change
C
HCC-LM2: onc.ch7.29 vs control
down-regulated up-regulated
Hallmark gene-sets
Enrichment score
INTERFERON GAMMA RESPONSE
INTERFERON ALPHA RESPONSE
PANCREAS BETA CELLS
MYOGENESIS
EPITHELIAL MESENCHYMAL TRANSITION
KRAS SIGNALING DN
BILE ACID METABOLISM
HCC-LM2: onc.ch17.67 vs control
down-regulated up-regulated
Hallmark gene-sets
INTERFERON GAMMA RESPONSE
INTERFERON ALPHA RESPONSE
E2F TARGETS
KRAS SIGNALING DN
Enrichment score
D
MDA-231: onc.ch7.29 vs control
down-regulated up-regulated
Hallmark gene-sets
INTERFERON GAMMA RESPONSE
INTERFERON ALPHA RESPONSE
EPITHELIAL MESENCHYMAL TRANSITION
COAGULATION
INFLAMMATORY RESPONSE
TNFA SIGNALING VIA NFKB
KRAS SIGNALING DN
MDA-231: onc.ch17.67 vs control
down-regulated up-regulated
Hallmark gene-sets
INTERFERON GAMMA RESPONSE
INTERFERON ALPHA RESPONSE
IL6 JAK STAT3 SIGNALING
INFLAMMATORY RESPONSE
TNFA SIGNALING VIA NFKB
PANCREAS BETA CELLS
KRAS SIGNALING DN
E
Control
onc.ch7.29
DAPI
Vimentin
MDA-MB-231
P < 8e−6
Vimentin mean intensity
Control
onc.ch7.29

Figure S4. Validation of function oncRNAs *In vivo* models of cancer progression. Related to Figure 4.
(A) Area under the curve (AUC) of the bioluminescence plots from the lung colonization assays with MDA-MB231 cell lines (left) and HCC-LM2 cell lines (right), corresponding with Fig 4B and 4D, respectively. *P* values were calculated using a one-tailed Mann-Whitney test. **(B)** Volcano plots of differentially expressed genes in MDA-MB231 cells overexpressing oncRNA.ch7.29 or oncRNA.ch17.67 compared to MDA-MB231 controls. The *P* values were calculated using DESeq2. The *P* value cut-off corresponds to a 10% FDR. **(C–D)** Informative iPage pathways associated with HCC-LM2 cells overexpressing oncRNA.ch7.29 or oncRNA.ch17.67 compared to controls (C) and MDA-231 cells overexpressing oncRNA.ch7.29 or oncRNA.ch17.67 compared to controls (D). **(E)** Immunofluorescence staining for vimentin (green) in control and onc.ch7.29 overexpressing breast cancer cell line, MDA-MB-231. Top panels show DAPI (blue) signals. Vimentin intensity for control cells and onc.ch7.29 overexpressing cells were quantified using the raw images (*n* = 14 and 26, respectively). One-tailed Mann–Whitney *U* test was used to compare measurements. Brightness of images were adjusted for visualization purposes only. Data are represented as mean ± SEM. Scale bar length corresponds to 40 um.

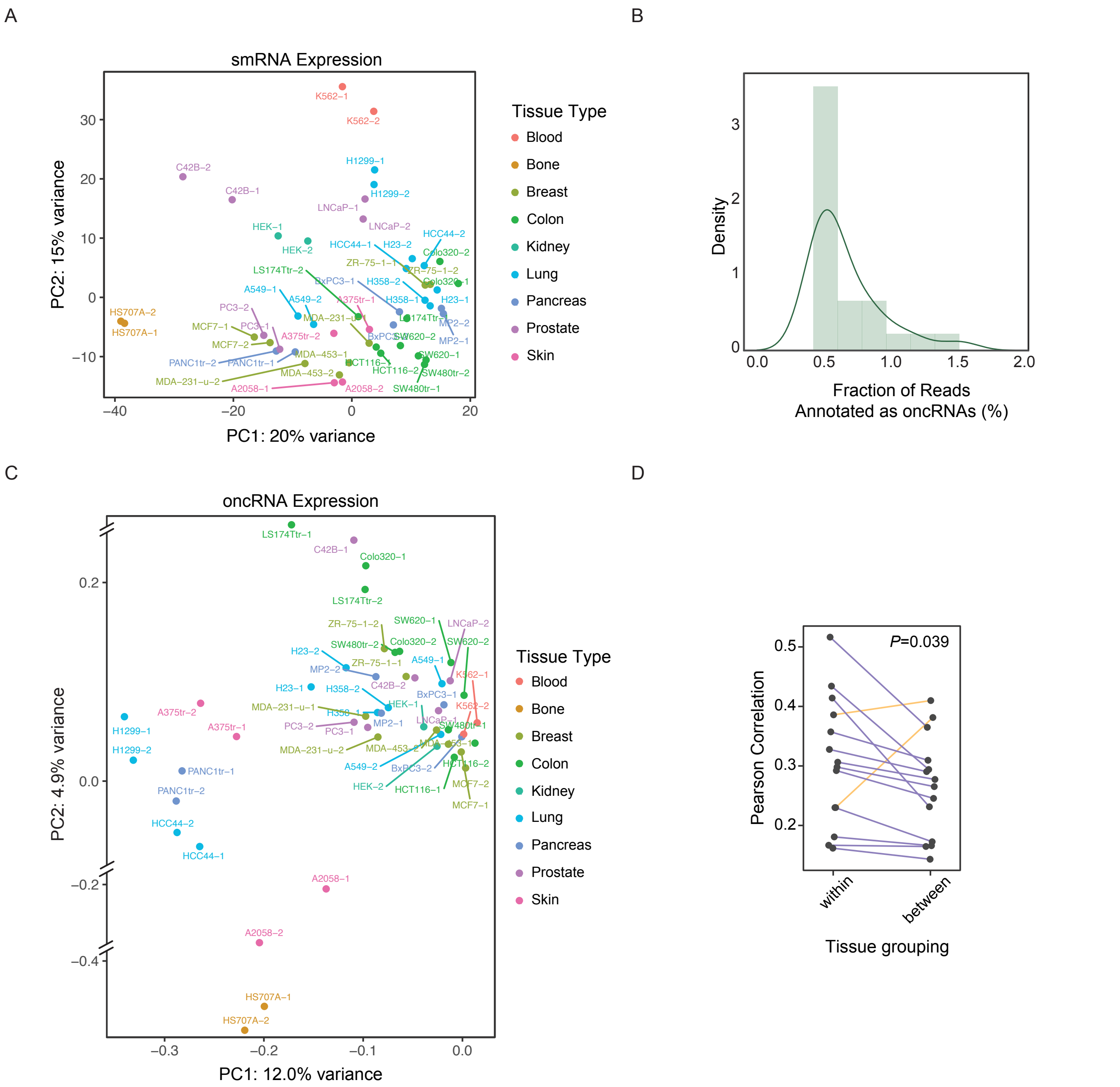

Figure S5. oncRNAs reflect cancer cell line identity in extracellular space. Related to Figure 5.

(A) PCA plot of the cell-free smRNA expression profiles of 25 cancer cell lines. Points are colored by the tumor type of the cell lines. **(B)** Density plot of the fraction of reads annotated as oncRNAs across all cancer cell lines in (A). **(C)** PCA plot of the cell-free oncRNA expression profiles in the cancer cell lines. Points are colored by the cell lines' corresponding tumor types. **(D)** Median Pearson correlation of oncRNA profiles between cell lines of the same tumor type (within) and cell lines of the same tumor type versus all other cell lines (between). Each connected pair of points consists of one reference tumor type. Tumor types with higher between-cancer tissue group correlations are colored orange, while tumor types with higher within group correlations are colored purple. Also reported is the *P* value calculated using a two-tailed paired Student's *t*-test.

A

		Missing	Overall
N			192
**Age, mean (SD)**		0	47.9 (10.9)
**T Stage, *n* (%)**	T1/T2	0	105 (54.7)
	T3/T4		67 (34.9)
	Undefined		20 (10.4)
**Node Status, *n* (%)**	Node+	0	89 (46.4)
	Node-		78 (40.6)
	Undefined		25 (13.0)
**Subtype, *n* (%)**	HER2+	0	12 (6.2)
	HR+HER2-		109 (56.8)
	TNBC		71 (37.0)
**pCR, *n* (%)**	0	0	141 (73.4)
	1		51 (26.6)
**RCB, *n* (%)**	0	0	53 (27.6)
	I		27 (14.1)
	II		79 (41.1)
	III		29 (15.1)
	Undefined		4 (2.1)
**Arm, *n* (%)**	Pac	0	105 (54.7)
	Pac + MK		25 (13.0)
	Pac + MK + Tras		10 (5.2)
	Pac + Pemb		50 (26.0)
	Pac + Tras		2 (1.0)
**oncRNA, *n* (%)**	High	0	37 (19.3)
	Mid		4 (2.1)
	Low		151(78.6)

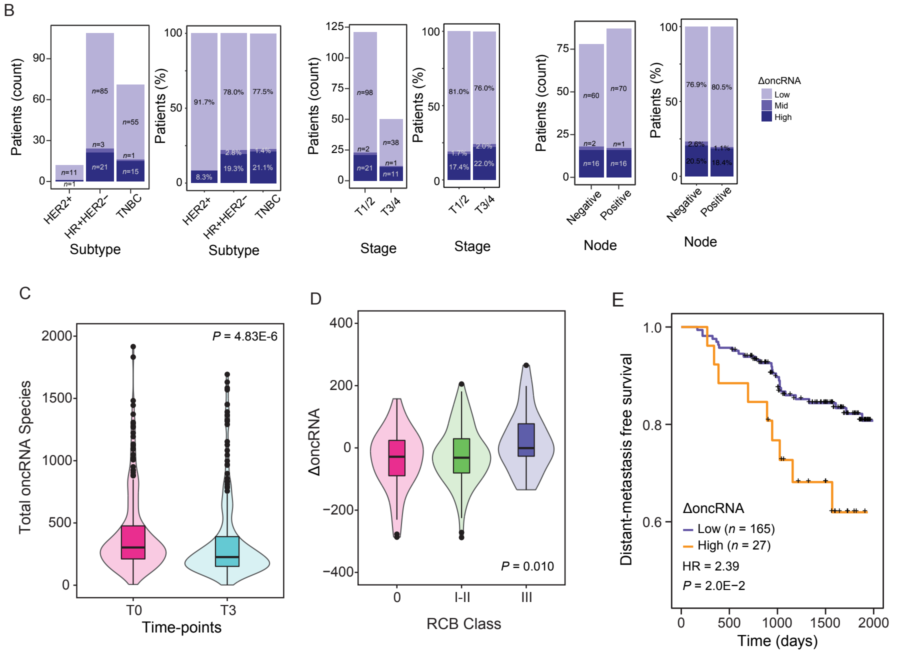

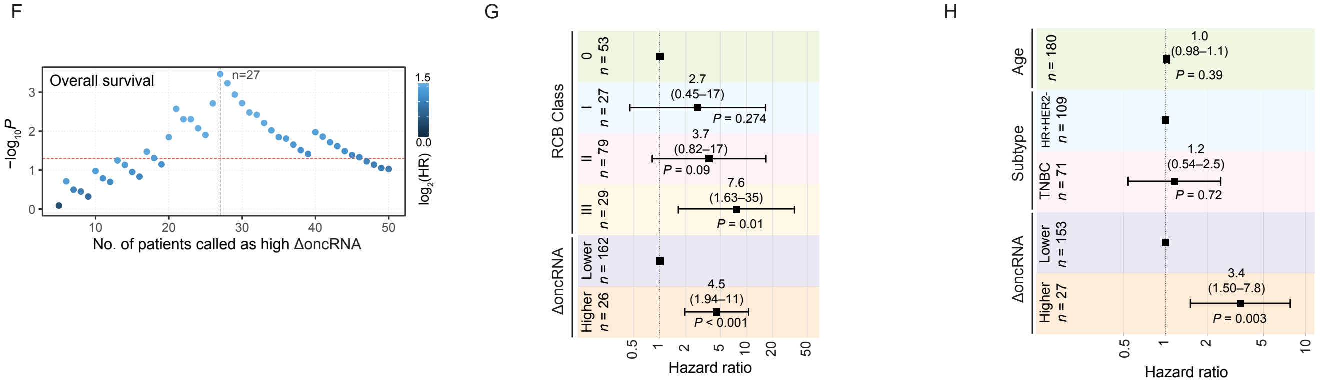

Figure S6. Analysis of residual oncRNA burden in the ISPY-2 trial cohort.

(A) Summary statistics of the ISPY-2 trial patient cohort (*n* = 192). Only patients with samples that passed our quality control filters for both time point 0 (prior to neoadjuvant chemotherapy) and time point 3 (prior to surgery) are included in this table. **(B)** Distributions of residual oncRNA burden (ΔoncRNA) levels among ISPY-2 patients, grouped by breast cancer subtype, tumor T classification, and node status. Shown are both the counts and normalized proportion of patients within each stratified ΔoncRNA level. **(C)** Number of oncRNA species detected in patient serum before (T0) and after (T3) neoadjuvant chemotherapy. *P* value was calculated using a one-tailed Wilcoxon test. Values are shown as violin plots and boxplots. The boxplots show the distribution quartiles, and the whiskers show the quartiles ± IQR (interquartile range). **(D)** ΔoncRNA of patients grouped by clinically determined residual cancer burden (RCB) class. RCB 0 indicates pathological complete response while RCB III indicates high residual cancer burden. *P* value was calculated using a one-way ANOVA test. Values are shown as violin plots and boxplots. The boxplots show the distribution quartiles, and the whiskers show the quartiles ± IQR (interquartile range). **(E)** Distant-metastasis free survival of patients grouped by ΔoncRNA. Also reported are the hazard ratio and *P* value based on a log-rank test. **(F)** Scatterplot of number of patients called as high ΔoncRNA versus resulting log-rank test $-\log_{10}$ *P* values using the cognate ΔoncRNA stratification. Points are colored by the resulting $\log_2$ hazard ratio. The ΔoncRNA threshold used for grouping high and low residual oncRNA burden in our reported survival analyses resulted in 27 high ΔoncRNA patients. **(G–H)** Forest plots of multivariate Cox proportional hazard model with ΔoncRNA and RCB class as covariates (G) and ΔoncRNA, subtype, and age as covariates (H). *P* values from the multivariate Cox analysis for each covariate are also included. HER2 positive samples were excluded due to small sample size, and samples with missing clinical data were omitted.

Project	Biofluid	Number of Samples
GSE112343	Bile	12
GSE113994	Plasma	212
	Serum	94
GSE121978	Urine	78
GSE123336	Saliva	87
	Serum	131
GSE128348	Urine	205
GSE128359	Plasma	123
	Stool	33
	Urine	47
GSE140069	Blood	105
GSE46579	Blood	69
GSE53439	Blood	2
	Serum	22
phs001258	Plasma	180
	Saliva	36
	Urine	203

Table S1. Publicly available datasets from the exRNA Atlas used to filter RNAs. Related to Figure 1.

Cancer	Precision	Recall	f1-Score
ACC	0.94	0.94	0.94
BLCA	0.81	0.79	0.80
BRCA	0.90	0.98	0.94
CESC	0.86	0.69	0.77
CHOL	1.00	0.86	0.92
COAD	0.80	0.89	0.85
DLBC	0.88	0.78	0.82
ESCA	0.96	0.65	0.77
HNSC	0.84	0.88	0.86
KICH	1.00	0.92	0.96
KIRC	0.91	0.97	0.94
KIRP	0.95	0.91	0.93
LAML	1.00	0.97	0.99
LGG	1.00	1.00	1.00
LIHC	0.99	0.93	0.96
LUAD	0.89	0.91	0.90
LUSC	0.72	0.81	0.76
MESO	0.94	0.88	0.91
OV	0.99	0.99	0.99
PAAD	0.94	0.83	0.88
PCPG	1.00	0.97	0.99
PRAD	1.00	0.97	0.98
READ	0.58	0.44	0.50
SARC	0.87	0.89	0.88
SKCM	0.98	0.99	0.98
STAD	0.89	0.89	0.89
TGCT	1.00	0.97	0.98
THCA	0.97	0.94	0.96
THYM	1.00	1.00	1.00
UCEC	0.89	0.93	0.91
UCS	1.00	0.82	0.90
UVM	1.00	0.94	0.97
**Accuracy**			0.91
**Macro-average**	0.92	0.89	0.90
**Weighted-average**	0.91	0.91	0.91

Table S2. Performance metrics of binary oncRNA XGBclassifier in tissue-of-origin classification. Related to Figure 1.

Performance metrics of the tissue-of-origin (TOO) XGBclassifier trained on binary oncRNA profiles and evaluated on the held-out dataset.

Cancer	Precision	Recall	f1-Score
ACC	0.94	1.00	0.97
BLCA	0.88	0.77	0.82
BRCA	0.90	0.98	0.94
CESC	0.82	0.79	0.80
CHOL	0.86	0.86	0.86
COAD	0.81	0.87	0.84
DLBC	0.75	0.67	0.71
ESCA	0.88	0.59	0.71
HNSC	0.79	0.89	0.84
KICH	1.00	0.92	0.96
KIRC	0.93	0.97	0.95
KIRP	0.97	0.97	0.97
LAML	0.97	0.97	0.97
LGG	1.00	1.00	1.00
LIHC	0.99	0.93	0.96
LUAD	0.84	0.88	0.86
LUSC	0.76	0.82	0.79
MESO	1.00	0.82	0.90
OV	0.99	0.99	0.99
PAAD	1.00	0.83	0.91
PCPG	1.00	0.97	0.99
PRAD	1.00	0.98	0.99
READ	0.58	0.47	0.52
SARC	0.88	0.96	0.92
SKCM	0.98	0.99	0.98
STAD	0.85	0.81	0.83
TGCT	1.00	0.97	0.98
THCA	1.00	0.93	0.96
THYM	1.00	1.00	1.00
UCEC	0.89	0.90	0.89
UCS	1.00	0.73	0.84
UVM	1.00	0.94	0.97
**Accuracy**			0.91
**Macro-average**	0.91	0.88	0.89
**Weighted-average**	0.91	0.91	0.91

Table S3. Performance metrics of XGB classifier trained on normalized oncRNA expression data. Related to Figure 1.

Performance metrics of the final tissue-of-origin (TOO) XGBclassifier trained on normalized oncRNA expression profiles (counts-per-million) and evaluated on the held-out dataset.

Cancer	Number of Samples
Breast carcinoma	106
Colorectal Cancer	106
Lung adenocarcinoma	232
Lung squamous cell carcinoma	109
Pancreatic ductal adenocarcinoma	146
Uterine corpus endometrial carcinoma	239

Table S4. Table of sample distribution across 6 cancer types from the Clinical Proteomic Tumor Analysis Consortium. Related to Figure 1.

Cancer	Precision	Recall	f1-Score
BRCA	0.55	0.99	0.71
CRC	0.85	0.93	0.89
LUAD	0.85	0.87	0.86
LUSC	0.82	0.85	0.83
PAAD	1.00	0.31	0.47
UCEC	0.98	0.95	0.96
**Accuracy**			0.82
**Macro-average**	0.84	0.82	0.79
**Weighted-average**	0.87	0.82	0.81

Table S5. Performance metric of TCGA trained XGBclassifier. Related to Figure 1.

Performance metrics of the tissue-of-origin XGBclassifier trained on binary oncRNA profiles from TCGA samples and evaluated on CPTAC samples.

Supplemental References

1. Culbertson, B. et al. A sense-antisense RNA interaction promotes breast cancer metastasis via regulation of NQO1 expression. Nat Cancer 4, 682–698 (2023).
